# White matter dissection and structural connectivity of the human vertical occipital fasciculus to link vision-associated brain cortex

**DOI:** 10.1038/s41598-020-57837-7

**Published:** 2020-01-21

**Authors:** Tatsuya Jitsuishi, Seiichiro Hirono, Tatsuya Yamamoto, Keiko Kitajo, Yasuo Iwadate, Atsushi Yamaguchi

**Affiliations:** 1 Department of Functional Anatomy, Graduate School of Medicine, Chiba University, 1-8-1 Inohana, Chuo-ku, Chiba 260-8670 Japan; 2 Department of Neurosurgery, Graduate School of Medicine, Chiba University, 1-8-1 Inohana, Chuo-ku, Chiba 260-8670 Japan; 3 Chiba Prefectural University of Health Sciences, 2-10-1 Wakaba, Mihama-ku, Chiba 260-0014 Japan

**Keywords:** Brain, Extrastriate cortex

## Abstract

The vertical occipital fasciculus (VOF) is an association fiber tract coursing vertically at the posterolateral corner of the brain. It is re-evaluated as a major fiber tract to link the dorsal and ventral visual stream. Although previous tractography studies showed the VOF’s cortical projections fall in the dorsal and ventral visual areas, the post-mortem dissection study for the validation remains limited. First, to validate the previous tractography data, we here performed the white matter dissection in post-mortem brains and demonstrated the VOF’s fiber bundles coursing between the V3A/B areas and the posterior fusiform gyrus. Secondly, we analyzed the VOF’s structural connectivity with diffusion tractography to link vision-associated cortical areas of the HCP MMP1.0 atlas, an updated map of the human cerebral cortex. Based on the criteria the VOF courses laterally to the inferior longitudinal fasciculus (ILF) and craniocaudally at the posterolateral corner of the brain, we reconstructed the VOF’s fiber tracts and found the widespread projections to the visual cortex. These findings could suggest a crucial role of VOF in integrating visual information to link the broad visual cortex as well as in connecting the dual visual stream.

## Introduction

The VOF is the fiber tract that courses vertically at the posterolateral corner of the brain. The VOF was historically described in monkey by Wernicke^1^ and then in human by Obersteiner^2^. It is re-evaluated by the advances in neuroimaging methods such as tractography, as a major association fiber tract to connect the dorsal and ventral visual cortex^3–6^. Previous tractography studies showed the cortical projections of the VOF fall in the dorsal (e.g., V3A, V3B, V3d, IPS-0) and the ventral visual areas (e.g., hV4, VO-1, VO-2) as well as in the lateral occipital cortex (e.g., LO-1, LO-2)^4,7,8^. However, there is no modern white matter dissection study to validate directly the results of VOF tractography.

Tractography using diffusion-weighted magnetic resonance imaging (DWI) has been widely used to estimate noninvasively connections between gray matter regions. This method, although elegant and elective, is prone to multiple artifacts due to “crossing, branching, merging, and termination” pitfalls^9,10^. It is also difficult to validate the data due to the lack of ground truth. In contrast, the modern white matter fiber dissection, although complex and time consuming, is a scientific procedure that could provide a thorough three-dimensional understanding of gray and white matter structures^11^. Many tracts and fasciculi were discovered by pioneer works of fiber dissection in neuroanatomy. However, fiber dissection lost its significance at the beginning of the twentieth century with the introduction of microtome and histological techniques. Klingler’s technique, a modern white matter dissection method, was developed in the 1930s, which is a modified method to facilitate the isolation of white matter tracts^12^. In this context, the fiber tracts and anatomical features in tractography studies have been validated by applying this dissection method^13,14^.

The dual visual stream hypothesis proposed the processing of visual information in the two distinct routes. The dorsal stream is involved in the perception of the spatial vision and vision-guided actions, while the ventral stream is related to identifying objects^15,16^. Previous studies reported the functional interaction between the dorsal and ventral visual stream^4,7^. For instance, saccade planning evokes cortical activity in both the dorsal (V3A/B, IPS0) and ventral visual areas (hV4, VO-1) in functional MRI (fMRI) study^17^. The dorsal visual cortex is strongly engaged by three-dimensional (3D) perception cooperating with the ventral visual cortex^18–20^. These functional findings suggest the significance of the interaction between the two visual streams, which is to be revealed by not only neuroimaging methods but also white matter dissection.

The results of neuroimaging studies, including tractography and fMRI, depend on the region of interest (ROI) of the structural MRI images. It means ROI selection influences the final interpretation of white matter pathways and brain functions^9^, therefore a common neuroscience framework for ROI selection is preferable. Brain parcellation is hard challenge due to the large number of regions and pathways, the remarkable individual variability in brain anatomy, the complex regional boundaries and interregional connectivity (NIH Connectome Coordination Facility). A variety of parcellation methods for visual cortex have been reported, which are based on cortical cytoarchitecture^21^, cortical folding patterns^22^, visual-field maps^23,24^, intrinsic functional organization^25^, and cortical myelination^26^. Although previous studies have attempted to delineate cortical areas mostly with a single feature, Glasser *et al*.^27^ defined distinct regions in the human cerebral cortex with a combination of brain-mapping techniques (cortical myelin content, cortical thickness, task-based fMRI, and resting-state fMRI) with semi-automated neuroanatomical approach. This HCP MMP1.0 (Human Connectome Project Multi-Modal Parcellation version 1.0), an updated map of the human cerebral cortex, would allow for neuroscientist in the fields of brain structure, function, and connectivity to work within a common neuroscience framework^27–29^. In this context, we analyzed the structural connectivity of VOF on the HCP multi-modal parcellation.

We first performed the white matter dissection in post-mortem human brains to validate the previous tractography data of the VOF, focusing on cortical projections in the two visual streams. Secondly, we analyzed the structural connectivity of the VOF with diffusion tractography based on HCP MMP1.0 atlas.

## Results

### White matter dissection of VOF in the lateral surface of the hemisphere

VOF is the most lateral fiber tract at the postero-lateral corner of the brain, running in a vertical direction (craniocaudally) posterior to the arcuate fasciculus (AF) and lateral to the inferior longitudinal fasciculus (ILF) and the inferior frontal occipital fasciculus (IFOF). The dorsal VOF’s cortical terminations consistently fall in the transverse occipital sulcus (TOS) and posterior intraparietal sulcus (IPS) in tractography^4^.

**Figure 1:**
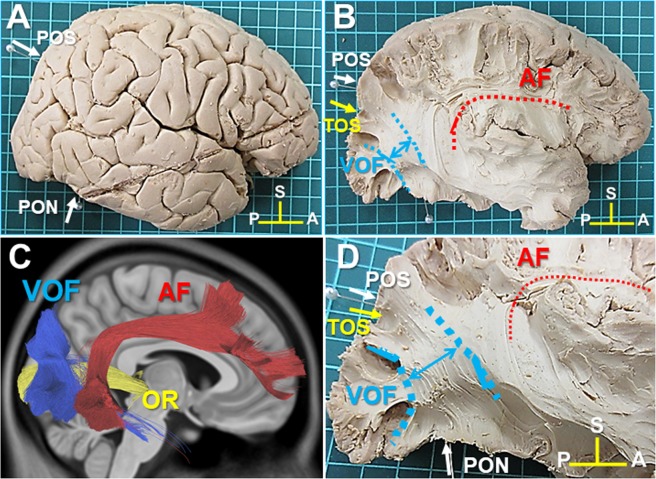
Lateral view of the right hemisphere (Sample #1). (**A**) The lateral view of right hemisphere after the removal of the meninges and vessels. (**B**) The lateral view of right hemisphere after the removal of the frontoparietal and temporal opercula around the insula to expose AF and VOF. (**C**) The trajectory of VOF (blue), AF (red), and OR (green) in diffusion-weighted tractography. (**D**) The magnification of the posterolateral corner of the brain to show the fiber bundles of VOF and AF. AF; arcuate fasciculus, OR; optic radiation, POS; parieto-occipital sulcus, TOS; transverse occipital sulcus, PON; pre-occipital notch. A; anterior, P; posterior, S; superior.

First, the representative anatomical landmarks were identified, including the parieto-occipital sulcus (POS), pre-occipital notch (PON), and TOS in the right hemisphere of brain sample #1 (Fig. 1A,B). Next, to expose the fiber bundles of the AF, we removed the fronto-parietal and temporal opercula around the insula (Fig. 1B). After the fiber bundles of the AF appeared at the posterior end of the insula, we continued to dissect toward the postero-lateral corner of the brain at this level and then found the lateral portion of VOF’s fiber bundles (Fig. 1B). The trajectory of the VOF courses in an oblique direction between the dorsal and ventral cortex at the postero-lateral corner of the brain (Fig. 1D), which is consistent with that of the VOF tractography (Fig. 1C).

### Cortical projections of the VOF in the dorsal and ventral visual stream

Recent tractography studies showed the cortical projections of the VOF fall in the dorsal (e.g., V3A, V3B, V3d, IPS-0) and the ventral visual areas (e.g., hV4, VO-1, VO-2) as well as in the lateral occipital cortex (e.g., LO-1, LO-2). Among them, the dorsal V3A/B and ventral hV4/VO-1 visual areas are the major cortical projection areas in tractography^4,7,8^.

**Figure 2:**
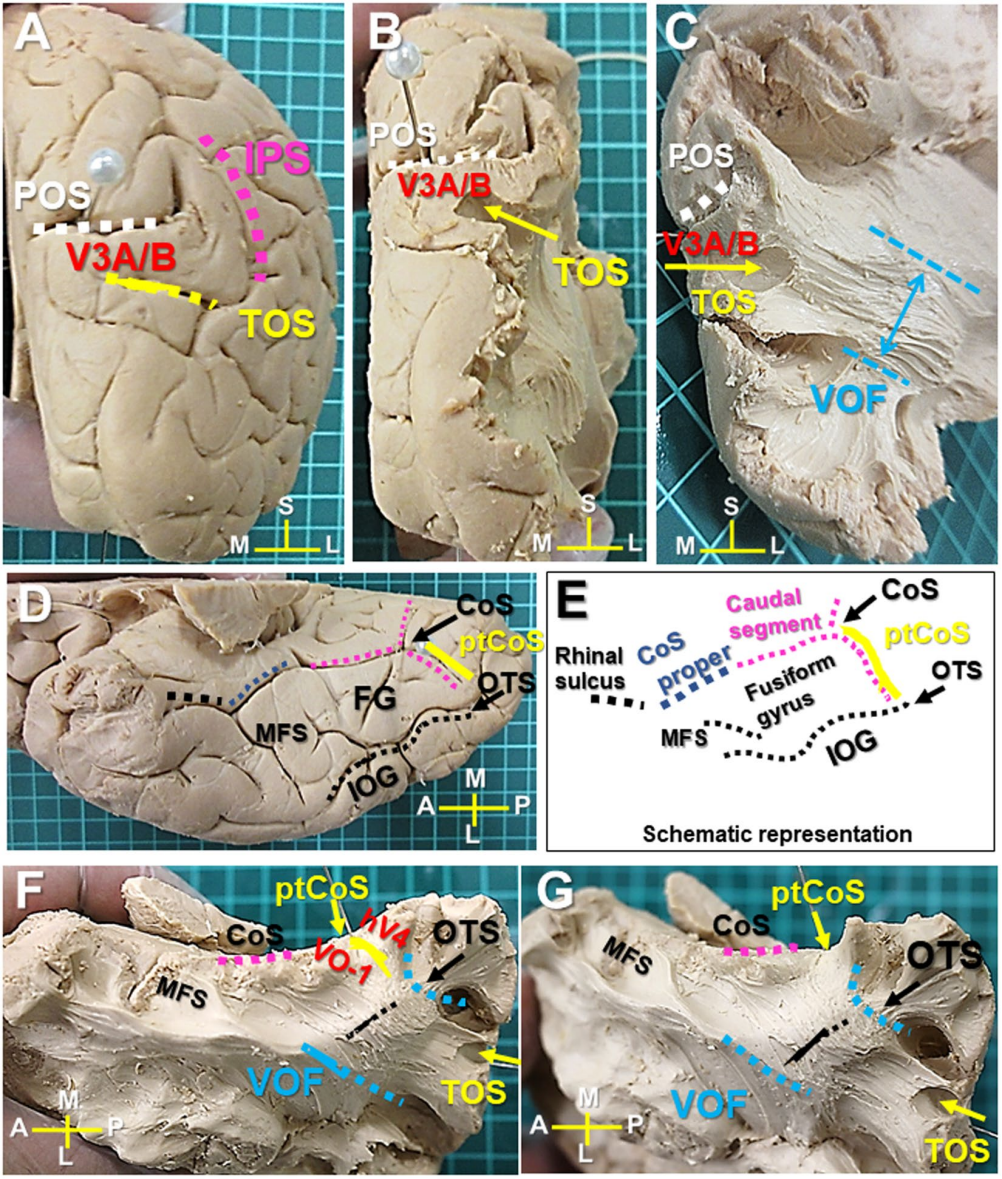
VOF’s cortical projections in the dorsal and ventral visual cortex (Sample #1). (**A**) Caudal view of the right hemisphere. TOS is located at the bottom of IPS inferior to POS. (**B**) Caudal view after removal of the cortex to expose the area around TOS. (**C**) Further dissection to expose the VOF’s cortical projections into TOS. (**D**) Ventral view of the right hemisphere with representative anatomical landmarks. (**E**) Schematic representation of ventral temporo-occipital cortex to show the CoS complex (Rhinal sulcus, CoS proper, and caudal segment), ptCoS, OTS, IOG, and fusiform gyrus (FG). (**F**) Ventral temporal lobe after removal of the cortex to expose the white matter around OTS and ptCoS. (**G**) Further dissection to expose the ventral VOF’s cortical projections in posterior fusiform gyrus and ptCoS. IPS; intraparietal sulcus, POS; parieto-occipital sulcus. CC; corpus callosum, CoS; collateral sulcus, ptCoS; posterior transverse CoS, FG; fusiform gyrus, MFS; mid-fusiform sulcus, IOG; inferior occipital gyrus, OTS; occipito-temporal sulcus, TOS; transverse occipital sulcus. A; anterior, L; lateral, M; medial, P; posterior, S; superior.

We first investigated the cortical projections of the VOF in the dorsal visual areas, especially those in V3A/B visual areas. V3A, originally reported to cross the transverse occipital sulcus (TOS)^30,31^, locates along the medial aspect of the TOS, while V3B abuts V3A laterally at the TOS^32^. These reports indicate the TOS is the landmark of V3A/B border. Since the intraparietal sulcus (IPS) often intersects the TOS in a T configuration, the TOS was identified at the bottom of the IPS as in Fig. 2A. We then tracked the VOF’s fiber bundles toward the TOS and observed they run into the TOS (the landmark of V3A/B border) (Fig. 2B,C). We also obtained similar results in the additional three samples (Supplementary Figs. S1D,G, S2D,G, S3D,G).

Next, we examined the cortical projections of the VOF in the ventral visual areas, especially those in the fusiform gyrus and hV4/VO-1 boundary. The previous tractography study showed the ventral VOF’s endpoints concentrate in the inferior occipital gyrus (IOG), inferior occipital sulcus (IOS), and posterior transverse collateral sulcus (ptCoS)^4^, which are shown in Fig. 2D,E. The occipito-temporal sulcus (OTS) runs antero-posteriorly separating IOG laterally from the fusiform gyrus (FG)^33^ (Fig. 2D). The collateral sulcus (CoS) consists of three separate sulcal segments (i) an anterior segment (rhinal sulcus), (ii) a middle segment (CoS proper), and (iii) a caudal segment (Fig. 2D,E). The CoS proper forms the lateral border of the posterior parahippocampal cortex^34^ as in Fig. 2D,E. The ventral VOF’s endpoints extend to the posterior mid-fusiform sulcus (MFS) anteriorly, the lateral portions of the posterior FG, and the posterior portion of the OTS, and they never cross CoS proper in tractography (Fig. 2E)^4^. The hV4/VO-1 boundary, one of the VOF’s major projection areas^7,8^, locates consistently in the posterior transverse collateral sulcus (ptCoS) in neuroimaging study^35^, indicating the ptCoS is a useful landmark for hV4/VO-1. Then, the ptCoS was identified at the branching point of the caudal segment of CoS as in Fig. 2D,E, which is consistent with previous report^31^. Using the ptCoS as the landmark for hV4/VO-1 boundary, we tracked VOF’s fiber bundles inferiorly into the ventral occipito-temporal lobe. After crossing the OTS, VOF’s fiber bundles appeared to split mainly to the posterior fusiform gyrus and partially to the ptCoS (Fig. 2F,G). The medial and anterior border of VOF’s fiber bundles in the ventral cortex seemed never to pass the CoS and MFS, respectively (Fig. 2F,G). We also obtained similar results in the additional three samples (Supplementary Figs. S1F,G, S2F,G, S3F,G).

### VOF’s cortical projections on the HCP MMP1.0 atlas

**Figure 3:**
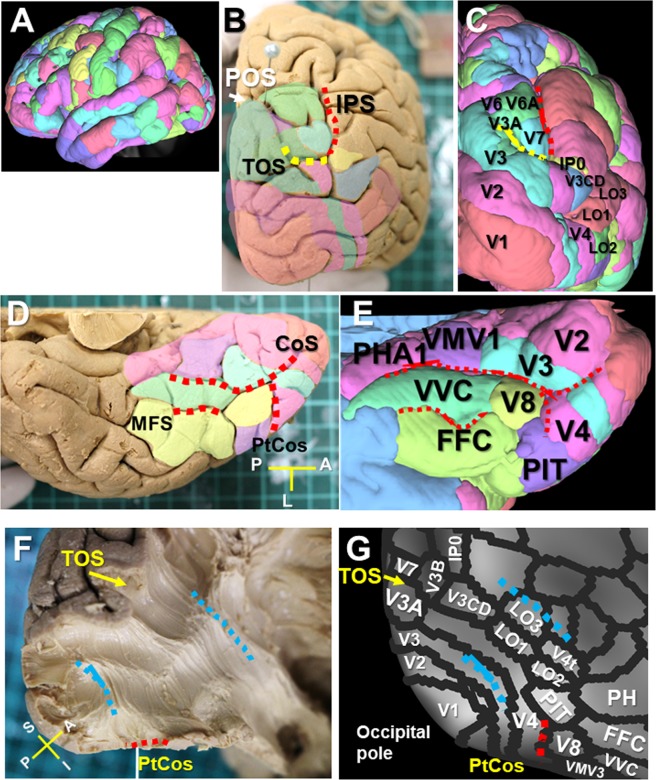
VOF’s cortical projections on the HCP MMP1.0 atlas. (**A**) Lateral view of HCP-1021 template overlaid with HCP MMP1.0 atlas. (**B**,**C**) Caudal view of a brain sample (**B**) and HCP-1021 template (**C**) with HCP MMP1.0 atlas. (**D**,**E**) Ventral temporal cortex of brain sample (**D**) and HCP-1021 template (**E**) with HCP MMP1.0 atlas. (**F**,**G**) Posterolateral corner of a brain sample (**F**) after dissection to expose the VOF’s fiber bundles running between ventral and dorsal visual stream, with corresponding HCP MMP1.0 atlas in a surface-based coordinate system (**G**). IPS; intraparietal sulcus, TOS; transverse occipital sulcus, POS; parieto-occipital sulcus. CoS; collateral sulcus, ptCoS; posterior transverse CoS, FG; fusiform gyrus, MFS; mid-fusiform sulcus, OTS; occipito-temporal sulcus. A; anterior, I; inferior, L; lateral, P; posterior, S; superior.

The HCP MMP1.0 atlas delineates180 cortical areas per hemisphere based on the degree of myelination, cortical thickness, and functional connectivity ^27^ (Fig. 3A). To show the anatomical relationship between the brain cortex and the HCP MMP1.0 atlas, we showed the image of a right hemisphere overlaid with the HCP MMP1.0 atlas (Fig. 3B–E). Figure 3F,G show the VOF’s fiber bundles appeared to travel between the dorsal parieto-occipital (e.g., V3A, V3B, V6, V6A, V7) and ventrolateral occipito-temporal cortex (e.g., V4, PIT, V8, FFC, VVC, LO1-3) on the HCP MMP1.0 atlas.

### Cortical areas on the HCP MMP1.0 atlas to reconstruct VOF’s fiber tracts

**Figure 4:**
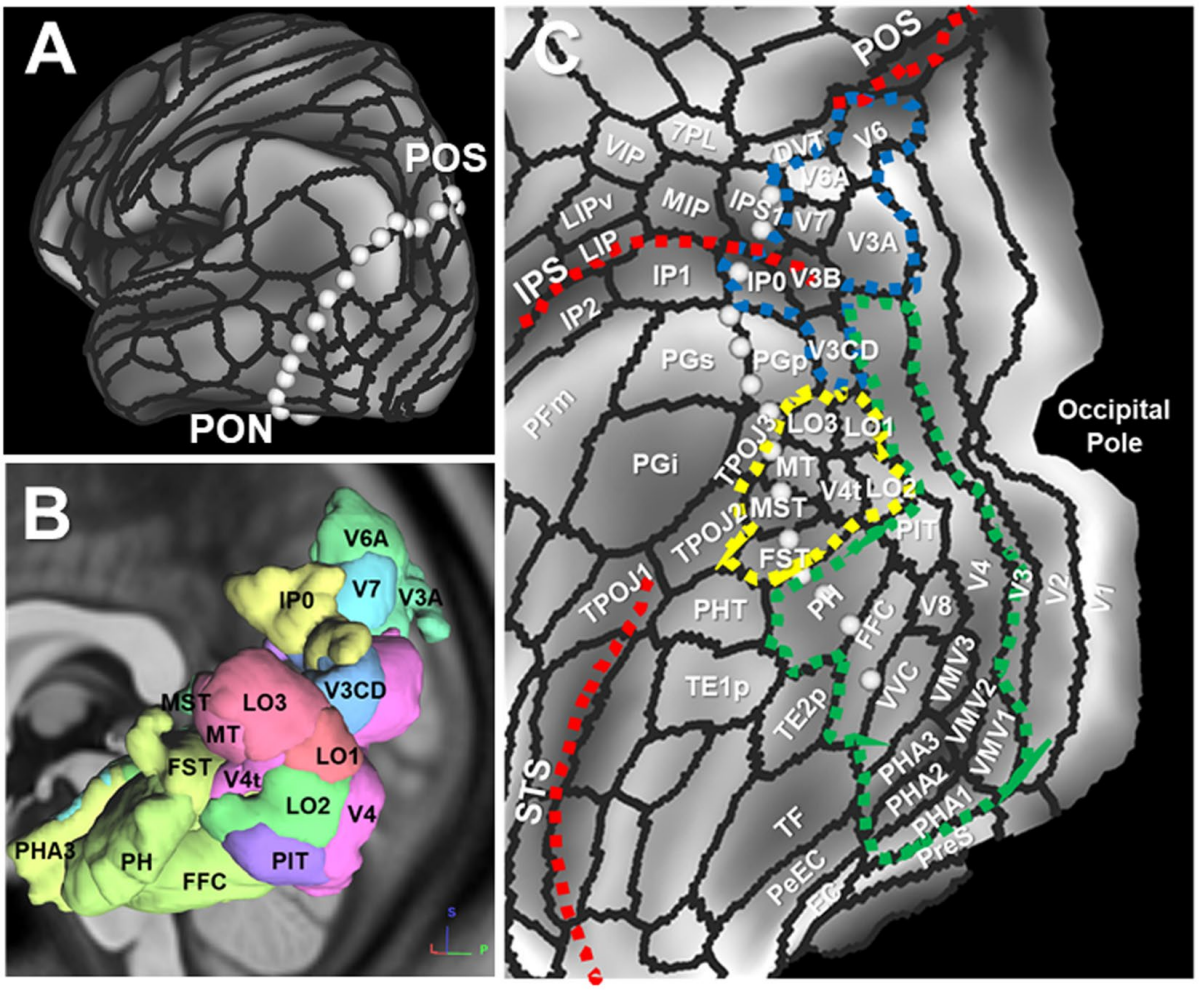
Vision-associated cortical areas on the HCP MMP1.0 atlas. (**A**) A line linking POS with PON on the inflated cortical surface of left hemisphere with HCP MMP1.0 atlas in a surface-based coordinate system. (**B**) Representative cortical areas of HCP MMP1.0 atlas on left posterolateral corner of the brain. (**C**) Three vision-associated cortical groups of the dorsal (blue dotted line), lateral occipital (yellow dotted line), ventral (green dotted line) cortex on the flattened cortical surface of left hemisphere. POS; parieto-occipital sulcus, PON; pre-occipital notch, IPS; intraparietal sulcus, STS; superior temporal sulcus.

The HCP MMP1.0 atlas delineates areas that are associated with auditory, somatosensory, visual, task-positive, or task-negative groups of areas in the resting state in a surface-based coordinate system termed “greyordinates”^27^. The vision-associated cortical areas are located mainly in the occipital lobe bordered by a line connecting POS (parietal-occipital sulcus) and PON (pre-occipital notch) as in Fig. 4A. Figure 4B,C indicate the vision-associated cortical areas on the posterolateral corner of the brain, including V3A, V3B, V3CD, V4, V4t, V6, V7, V8, IP0 (intraparietal 0), LO1-3 (lateral occipital 1-3), MT(middle temporal), MST (medial superior temporal), FST (fundus of the superior temporal sulcus area), PIT (posterior inferior temporal), VMV1-3 (ventromedial visual area 1-3), PHA1-3 (parahippocampal area 1-3), FFC (fusiform face complex), and VVC (ventral visual complex). To reconstruct VOF’s fiber tracts by a two-ROI approach, we selected two ROIs from three groups of vision-associated cortical areas, consisting of dorsal parieto-occipital (blue dotted line), lateral occipital (yellow dotted line), and ventral group (green dotted line) (Fig. 4C).

### Structural connectivity of VOF in the HCP-1021 template

**Figure 5:**
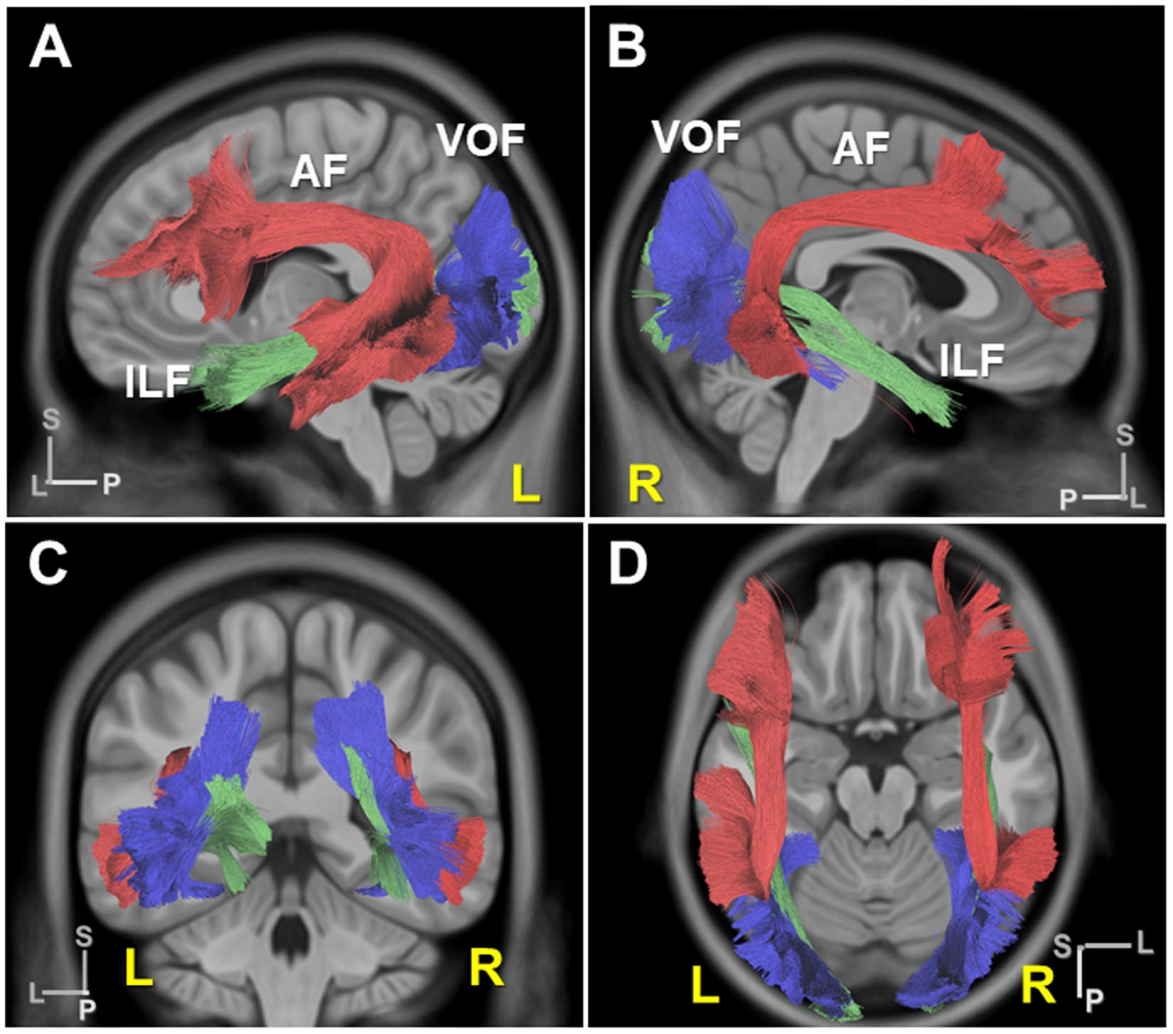
VOF tractography in the HCP-1021 template. (**A**,**B**) Lateral view of VOF’s trajectory (blue) in the HCP-1021 template in left (**A**) and right (**B**) hemisphere with AF (deep red) and ILF (deep green). (**C**,**D**) Coronal and horizontal view of VOF’s trajectory in the HCP-1021 template. AF; arcuate fasciculus, ILF; inferior longitudinal fasciculus. L, left; R, right; A; anterior, L; lateral, P; posterior, S; superior.

To reconstruct VOF’s fiber tracts, we first used HCP-1021 template averaged from a total of 1021 subject diffusion MRI datasets from the Human Connectome Project^36^. This template is explained in the “Experimental procedure” below. A previous white matter dissection study showed the inferior longitudinal fasciculus (ILF) is medial to the VOF within the sagittal stratum of Sachs (SSS)^37^, which is consistent with our dissection result. From our anatomical observations, the VOF is the bundle of fiber tracts to course craniocaudally between the dorsal parieto-occipital and the occipito-temporal cortex, laterally to ILF. Based on this criteria, we obtained the candidate tracts of the VOF in tractography using two ROIs from vision-associated cortical areas of HCP MMP1.0 atlas (Fig. 4C). Then only the tracts lateral to ILF were extracted to generate VOF tractography. The generated VOF trajectory courses posteriorly to the AF and laterally to the ILF at the postero-lateral corner of brain (Fig. 5A–D).

### Structural connectivity of the VOF in individual brains

**Figure 6:**
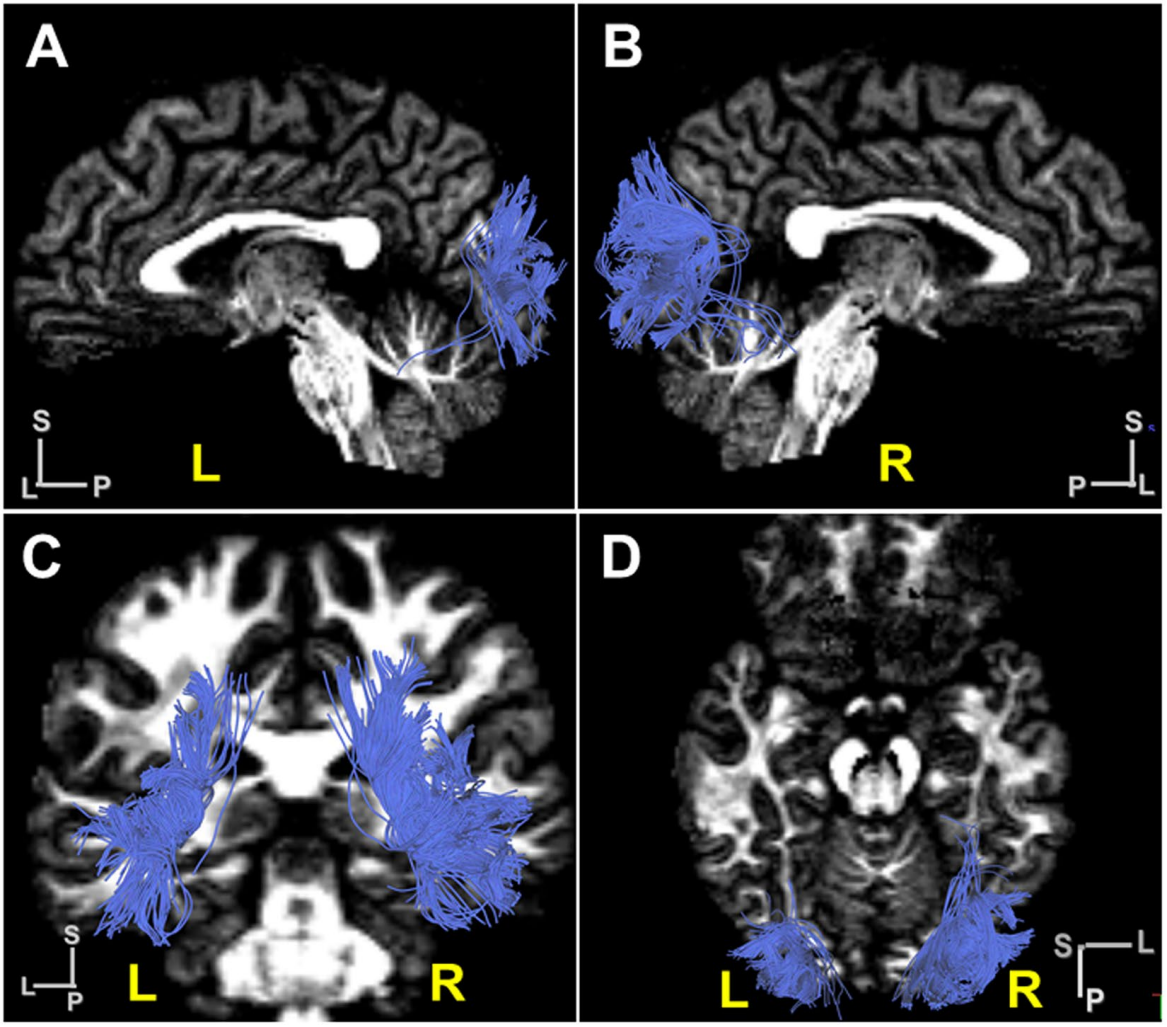
VOF tractography in a representative subject brain. (**A**,**B**) Lateral view of VOF’s trajectory in a subject brain (ID #102614) in the left (**A**) and the right (**B**) hemisphere. (**C**,**D**) Coronal and horizontal view of VOF’s trajectory in a subject brain (ID #102614). L, left; R, right; A; anterior, L; lateral, P; posterior, S; superior.

Next, we reconstructed VOF’ fiber tracts by a subject-based approach using ten healthy unrelated samples from the HCP dataset. Figure 6A–D show the VOF tractography in a representative subject (ID#102614), which appear similar to those in the HCP-1021 template.

**Figure 7:**
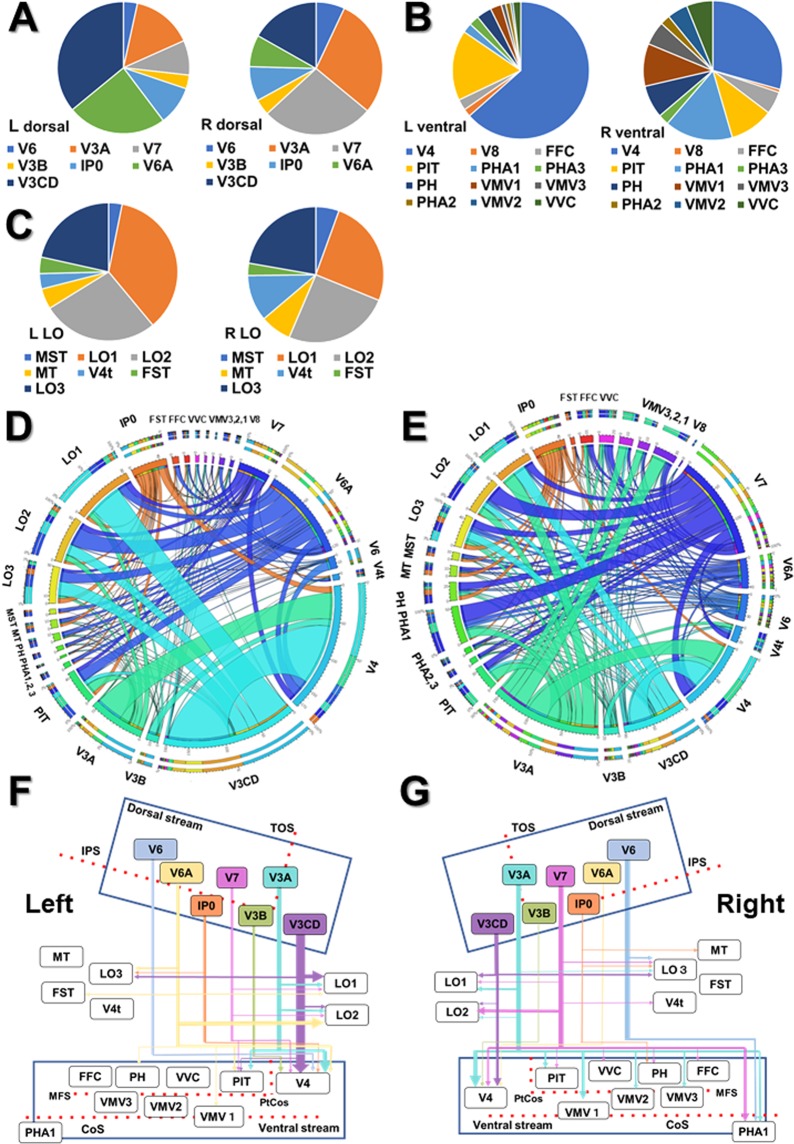
Endpoint analysis, Connectogram, and Schematic diagram for the connectivity patterns of VOF in individual brains. (**A**–**C**) The ratio (%) of cortical areas in which VOF’s cortical endpoints fall on dorsal (**A**), ventral (**B**), and lateral occipital (**C**) visual cortex in the left and the right hemisphere, respectively. The data is the average of 10 subjects. Dorsal, dorsal visual cortex; Ventral, ventral visual cortex; LO, lateral occipital visual cortex; L, left; R, right. (**D**,**E**) Connectograms indicating the connectivity patterns of VOF in the left (**D**) and the right (**E**) hemisphere, respectively. Circular color maps detail the scale for each connection. The outermost two rings show the various cortical areas of the HCP MMP 1.0 atlas. The length of arc reflects the count of tracts terminating within each cortical area. The ribbon size represents the computed degrees of connectivity (i.e., the number of tracts) between segmented brain regions. The data is the average of 10 subjects. MT, middle temporal; PHA, parahippocampal area; PIT, posterior inferior temporal; LO, lateral occipital; IP0, intraparietal area 0; FFC, fusiform face complex; VVC, ventral visual complex; VMV, ventromedial visual area. The original versions of these connectograms are shown in the Supplementary Figs. S7 and S8. (**F**,**G**) Schematic diagrams of structural connectivity for VOF’s fiber tracts in the left (**F**) and the right (**G**) hemisphere. The arrow size represents the computed degrees of connectivity (i.e., the number of tracts) between segmented brain regions. TOS, transverse occipital sulcus; IPS, intraparietal sulcus; CoS, collateral sulcus; ptCoS, posterior trasverse CoS; MFS, middle fusiform sulcus.

To investigate in which cortical area the VOF’s endpoints project, we then performed the endpoint analysis in individual brains on the HCP MMP1.0 atlas. Graphs in Fig. 7A shows the prominent endpoint projections to V3CD (35.2%), V6A (24.8%), V3A (14.8%), IP0 (9.5%), and V7 (8.5%) on the left dorsal visual stream, and those to V3A (29.4%), V7 (26.5%), V3CD (16.7%), IP0 (7.8%), and V6A (7.4%) on the right dorsal visual stream. Graphs in Fig. 7B show the remarkable endpoint projections to V4 (62.0%) and PIT (16.2%) on the left ventral visual stream, and those to V4 (26.5%), VMV1 (11.4%), PHA1 (10.2%), and PIT (5.1%) on the right ventral visual stream. Graphs in Fig. 7C show the endpoint projections to LO1 (35.7% and 23.4%), LO2 (27.4% and 25.7%), and LO3 (21.5% and 23.6%) in lateral occipital visual cortex, respectively.

**Figure 8:**
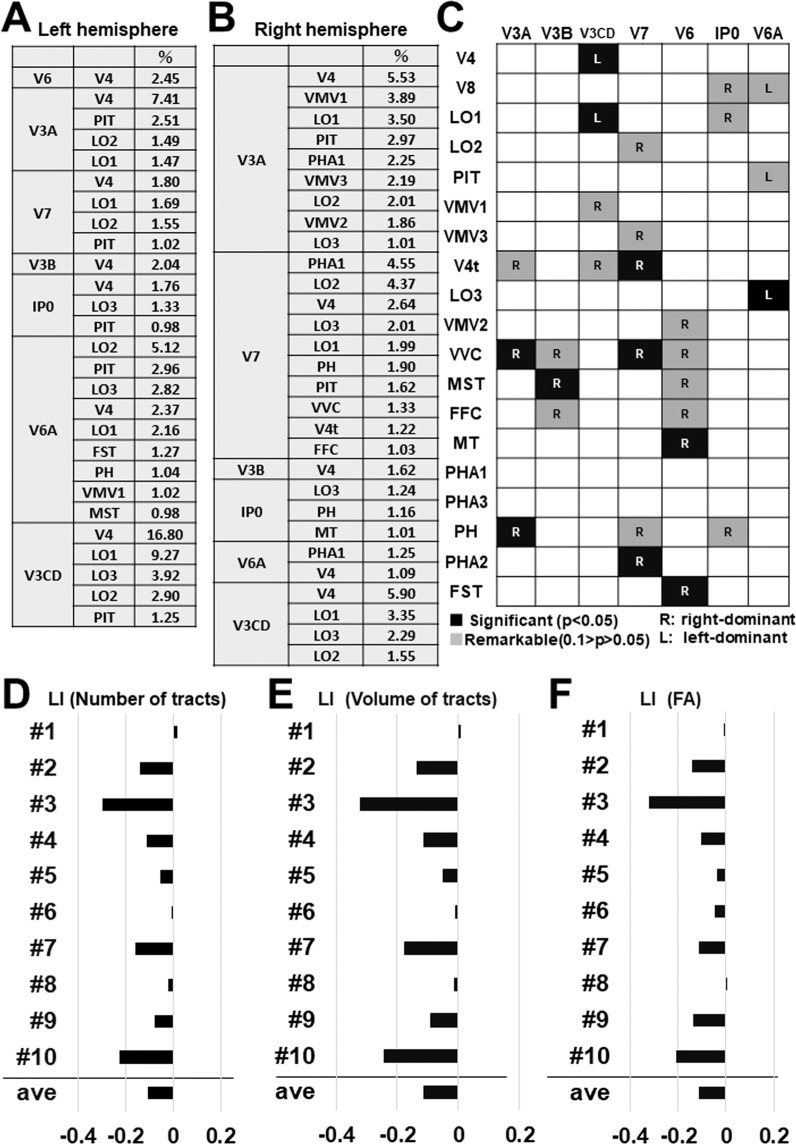
Ratio and laterality for the number, the volume, and the FA of VOF’s fiber tracts. (**A**,**B**) The table indicates the ratio (%) for the number of tracts to connect each combination of the cortical area in left and right hemisphere, respectively. The data is the average of 10 subjects. (**C**) The laterality for the number of tracts to connect each combination of the cortical area. The data is the average of 10 subjects. Darker colors indicate the strength of connection. R; right-ward lateralization, L; left-ward lateralization. (**D**–**F**) Graphs indicate the laterality index (LI) for the total number (**D**), the total volume (**E**), and the total FA (fractional anisotropy) (**F**) of VOF’s fiber tracts in individual brains (subject #1–10).

Connectogram^38,39^ is a way to visualize the brain’s connections at a glance, which can assess white matter fiber pathways between brain regions by measuring fiber bundle properties (e.g., the number of fiber tracts). We used the connectogram to map and interpret the overview of complex VOF’s connections (Fig. 7D,E). Then, to focus on the role of VOF to interconnect the dual visual stream, we drew schematic diagrams of the VOF’s connection patterns between the dorsal and ventral visual steam (Fig. 7F,G). The connectograms (Fig. 7D,E) represent the structural connectivity of VOF derived from average data of 10 individual samples in the left and right hemisphere, respectively. An interesting feature of the connectograms is the asymmetry in the connectivity patterns between the left and right hemisphere. The connectivity patterns in the right hemisphere appeared relatively variable compared with the left hemisphere. Schematic diagrams (Fig. 7F,G) show the VOF’s structural connectivity, emphasizing on the dual visual stream in the left and right hemisphere, respectively. The line size reflects the connectivity strength (i.e., number of tracts) of each fiber tract. The combination of these results showed the streamlines connecting V3CD with V4 appeared to be major in the left hemisphere, while those from V3A, V3CD, V6, and V7 were projected broadly to the ventral stream in the right hemisphere. In addition, lateral occipital visual areas (LO1, LO2, LO3) receive projections from V3CD, V6A in the left hemisphere and V7, V3CD, V3A in the right hemisphere, respectively. We observed a similar tendency in the analyses of HCP1021 template (Supplementary Fig. S4). The tables in Fig. 8A,B indicate the average ratio for the number of tracts comprising each VOF’s connection in left and right hemisphere in individual brains, respectively. The prominent fiber tracts in the left are those connecting V3CD-V4, V3CD-LO1, V3A-V4, and V6A-LO2, while those in the right are connecting V3A-V4, V3CD-V4, V7-LO2, and V7-PHA1. These results quantified the findings in the connectograms and schematic diagrams in Fig. 7D–G. The table in Fig. 8C shows the laterality for the number of tracts in individual brains comprising each VOF’s fiber tract derived from Supplementary Table 1, suggesting the relative rightward lateralization in the number of fiber tracts. The laterality index of the volume and FA (fractional anisotropy, i.e., the integrity of white matter) for each VOF’s fiber tract were shown in Supplementary Tables 2–4. Graphs in Fig. 8D–F indicate the laterality index for the total number, volume, and FA of tracts for each subject (#1-#10), suggesting the slightly rightward lateralization for not only quantitative (i.e., the number and volume of tracts) but also qualitative (i.e., the integrity of white matter) connectivity.

## Discussion

### White matter dissection and structural connectivity of the VOF

The aims in the present study were: 1) to validate the previous tractography data of the VOF by white matter dissection focusing on the cortical projections in the two visual streams, and 2) to analyze the structural connectivity of the VOF using the tractography based on the HCP MMP 1.0 atlas.

First, we performed the white matter dissection to validate the tracking data of the VOF previously reported^4,7^. Based on a strong relationship between the cortical folding and brain function^32,40^, we used the gyri and sulci as anatomical landmarks for dissection. In the dissection, the major dorsal VOF’s fiber bundles appeared to run into TOS (transverse occipital sulcus) where the V3A/B border is located, indicating VOF’s cortical projections could fall in V3A/B visual areas (Fig. 2A and Supplementary Figs. S1D, S2D, S3D). The ventral VOF’s fiber bundles appeared to run mainly in the posterior fusiform gyrus and partially to ptCoS (the boundary of VO-1/hV4) (Fig. 2F,G and Supplementary Figs. S1G, S2G, S3G). The anterior and medial border of the VOF in the ventral cortex appeared never to pass MFS and CoS, respectively (Fig. 2E–G). These results are almost consistent with the previous tractography data, suggesting the VOF could connect the dorsal and ventral visual cortex^4,7^.

There has been some controversy over the definition of VOF. Wernicke^1^ originally described the VOF as “the *senkrechte Occipitalbündel* (vertical occipital bundle)” on sagittal slices of the monkey^4^. Our dissection showed the major dorsal cortical projections appear to run into TOS, which suggests the major part of dorsal VOF’s fiber bundles could terminate in the occipital lobe. Others have used less precise definitions of the VOF and described tracts terminating in the parietal cortex and anterior temporal cortex as VOF. Recently Bullock *et al*.^41^ clearly present a full anatomical delineation of these major dorso-ventral connective white matter tracts, including the VOF, pArc, TP-SPL, and MdLF. They exclusively restricted the VOF to the occipital white matter. They showed the VOF connects the dorsal and ventral streams within the occipital lobe, although all three tracts share some endpoints in the occipitoparietal arc.

Secondly, we analyzed the structural connectivity of the VOF to link the vision-associated cortical areas on the HCP MMP1.0 atlas. Based on the criteria the VOF courses in a vertical direction (craniocaudally) between the dorsal parieto-occipital and ventrolateral occipito-temporal cortex laterally to the ILF, we reconstructed the VOF’s fiber tracts and examined the structural connectivity (Figs. 5 and 8).

### Structural connectivity of VOF in tractography

One noticeable point in our VOF tractography study is the striking connection linking the dorsal stream (V3A, V3CD, V6) with ventral stream (V4, FFC, VVC, VMV2, PIT), consistent with a previous report^42^. V3A is implicated in motion-selective visual perception with high motion and contrast sensitivity in the central visual field^23,29,30,43^, while V3CD integrates object detail information to create the whole image for object recognition. On the other hand, V4 reportedly plays a role in encoding texture, patterns, form, and color perception, integrating object and pattern recognition^23,29,30,43,44^. Ventral temporal occipital cortex (FFC, PIT, VMV1-3, VVC) shows a considerable functional heterogeneity from early visual processing to higher-level domain-specific processing. It harbors a strong relation to the visual language processing system including VWFA (visual word form area) in the left and face-selective regions in the right hemisphere^45^. Recently Kay *et al*.^46^ reported the VOF specifically connects VWFA and FFA with the IPS (intraparietal sulcus) by the combination of tractography and fMRI. These findings raise the possibility that VOF could integrate the various information related to object recognition (including word and face), location, and motion through the dual visual stream.

We also found the cortical projections of VOF to the lateral occipital visual area (e.g., LO1, LO2, LO3, V4t) from the dorsal visual stream (e.g., V3CD, V3A, IP0, V7). LO1 preferentially activates in response to orientation-selective and boundary information, while LO2 shows preferential retinotopic activation to encode information about the shapes of stimuli^29,47,48^. LO3 is an important hub between the dorsal and ventral streams to integrate, encode, and process detail, motion, and shape information^29,49^. Taken together, these results support the VOF could link the broad vision-associated cortex, integrating a variety of information about the face, body, word, shape, texture, color, orientation, location, and motion of objects.

Takemura *et al*.^7^ reported the visual field maps located on the lateral side (hV4, VO-1, and LO-map) receive larger VOF projections. Our results showed LO-map receive relatively small portion of VOF projections mainly from V3CD and V3A (Fig. 7C–E). One reason for this discrepancy is due to the difference in the definition of the visual field areas between Takemura *et al*.^7^ and Glasser *et al*.^27^.

### Laterality in the structural connectivity of VOF

We observed the slightly rightward lateralization in the number, volume, and FA (fractional anisotropy) of VOF’s fiber tracts. In addition, the variation in the connectivity patterns appeared relatively rich in the right compared with the left hemisphere. Since non-dominant hemisphere (usually right) is determinant for visuospatial cognition^50^, the rightward lateralization of VOF’s connectivity could relate to visuospatial cognition, including awareness, perception, and representation of space thorough a variety of connectivity. However the number of subjects is small, the conclusion on the laterality is limited.

### ROIs to reconstruct VOF’s fiber tracts in tractography

There is a variation in the description of VOF’s trajectory in previous tractography studies. We here focus on the ROIs used to reconstruct VOF’s fiber tracts in those studies. The major neuroanatomists agree on VOF’s ventral terminations in the fusiform gyrus and the inferior occipital and temporal gyrus^4^. Yeatman *et al*.^4^ then used a ROI of ventral occipito-temporal cortex including the fusiform gyrus, inferior temporal gyrus, and lateral occipital regions. From the ventral occipito-temporal fiber group terminating within 2 mm of the ventral surface, the vertical fibers that are spatially separated and posterior to the arcuate fasciculus were extracted as VOF. Takemura *et al*.^7^ focused on the posterior portion of VOF and identified VOF as the set of fascicles passing through two axial waypoint ROIs, located 3 and 14 mm above the dorsal edge of hV4. They showed the major cortical endpoints of VOF fall in V3A/B of the dorsal visual stream and in hV4/VO-1 of the ventral visual stream. Wu *et al*.^6^ reconstructed VOF by three seed regions (the fusiform gyrus, inferior occipital and temporal gyrus). With a ROI mask placed on the upper portion and a ROA drawn anteriorly to avoid other fiber contamination, they showed VOF connects the inferior parietal with the lower temporal and occipital lobe. We used this method in Fig. 1C. Keser *et al*.^5^ used multiple ROI tracking method to reconstruct VOF manually with the neuroanatomy guidance. They demonstrated VOF connects lateral occipital, posterolateral parietal, and posterosuperior temporal cortex using ROIs in the occipital lobe and parieto-occipital lobe junction and a ROA to avoid contamination of IFOF and ILF. Panesar *et al*.^51^ used the Automated Anatomical Labeling atlas (AAL) to select cortical seeding regions and generated VOF fibers using ROIs of three occipital gyri, including superior occipital gyrus (SO), middle occipital gyrus (MO), and cuneus (Cu), with a rectangular axial ROI to select only dorsal-ventrally passing fibers. They called this method an atlas based approach along with manually placed ROIs and a ROA. Briggs *et al*.^42^ delineated the boundaries of the VOF utilizing the cortex areas of the HCP MMP1.0 atlas, which we employed in Figs. 5 and 6. Taken together, the previous studies reconstructed the VOF’s fiber tracts with a variety of ROIs, including 1) manual plane ROIs (axially or coronally), 2) cortical ROIs built-in atlas (e.g., gyrus in AAL), 3) two waypoint axial ROIs, by anatomical guidance with or without a ROA. Then the only fiber tracts passing the selected ROIs craniocaudally, posterior to AF and laterally to ILF, were selected as VOF. Since Takemura *et al*.^7^ focused on the posterior portion of VOF, the description of anterior portion would be limited. As mentioned, the trajectories of VOF with manual ROIs and a ROA are sensitive to the positioning of them. The cortical endpoints in the previous studies appear to be variable, including those to the dorsal visual (V3A, V6, V6A) and ventral visual cortex (VVC, FFC). This is due to the positioning of the upper ROIs and an anterior ROA, which affect the dorsal and ventral cortical endpoints respectively^5,6,51^. Since we selected ROIs from vision-associated cortex on HCP MMP1.0, the cortical projections in the present study can cover over the dorsal and ventral visual cortex as well as the lateral occipital cortex. While we were preparing for this paper, Schurr *et al*.(2019) ^52^ reported an automatic procedure for VOF identification by integrating diffusion MRI tractography with quantitative T1 mapping, to clarify the anterior extent of VOF’s projection. They showed the anterior extent of VOF could be close to the posterior end of the MFS, which is compatible with our dissection result. They also pointed out the trajectory of VOF by waypoint ROIs method is restricted to the occipital white matter and tends to cover more lateral regions (V3B, LO1, and LO2), which we can agree with. The advantages of the method using HCP MMP1.0 in tractography are: 1) high affinity with functional analyses, 2) feasible to conduct the endpoint analysis, and 3) applicable for automatic analyses based on the common neuroscience framework. However, the anatomical information of the target tract is required for this method, including approximate projection areas in the cortex and the interaction with proximal tracts and structures. Therefore, we can apply this method only to the anatomically well-described fiber tracts.

## Conclusion

In summary, our results in the brain dissection support the previous tractography studies that VOF interconnects the dorsal and visual stream^4,7^. Furthermore, the analyses of the structural connectivity with diffusion tractography suggest VOF could integrate visual information connecting the broad visual cortex as well as the dual visual stream.

### Limitation

Port-mortem dissection allows us to visualize the white matter fibers particularly in depth, orientation, and relationships to gray matter structures. This suffers from drawbacks such as the highly observer-dependent definition of areal borders and identification of fiber bundles. The demonstration of one fiber system often results in the destruction of other fiber systems^53,54^. On the other hand, diffusion tractography has strongly contributed to our knowledge about fiber pathways by clarifying cortical connectivity patterns of white matter tracts. It suffers from several drawbacks. It is prone to multiple artifacts due to “crossing, branching, merging, and termination” pitfalls^9,10^. It is unable to demonstrate cortical connectivity accurately due to partial volume effects in voxels prone to tissues boundaries^55,56^.

## Experimental procedure

### White matter dissection

Five normal cerebral hemispheres (three right sides and two left sides) from human cadavers (age range 67–81 years) donated to the Chiba University were studied. White matter dissection was performed according to a modified Klingler’s technique^12,57^ as previously described^58^. After fixed in 10% formalin solution for at least 40 days, brains were washed under running water for several hours to remove the formalin. The pia mater, arachnoid membrane, and vessels of the specimens were carefully removed, and the hemispheres were frozen at −15 °C for 7 days. The specimens were allowed to thaw and then stored in the refrigerator once more for 5 days, as this protocol facilitates dissection. Major anatomical landmarks were identified with needle pins before dissecting the brain. The specimens after thawing were dissected in a stepwise manner from the lateral surface to the medial surface under the magnification loupe (x3.0) with wooden spatulas.

Photographs were taken at each step of the dissection to demonstrate the anatomical architecture. The digital photographs in this study were edited using Powerpoint (Microsoft Corporation) for white balance, contrast, sharpness with the images closely resembling the actual fiber tract architecture during the dissections.

### Ethics

Informed consent for cadaver use for research and education purposes was acquired from the members of the Whole-Body Donation Registry at Chiba University and their families. The protocol was approved by the Research Ethics Committee of Chiba University School of Medicine. This study was carried out in accordance with the “Guidelines for Cadaver Dissection in Education and Research of Clinical Medicine” by Japan Surgical Society and Japanese Association of Anatomists.

### The Human Connectome Project (HCP) dataset

The diffusion dataset in the present study was provided by the Human Connectome Project^36^ (http://humanconnectome.org) and WU-Minn Consortium (Principal Investigators: David Van Essen and Kamil Ugurbil; 1U54MH091657).

### HCP 1021 template

The HCP-1021 template^36^ was averaged from a total of 1021 subjects’ HCP data from the WU-Minn HCP Consortium (Q1–Q3, 2014) and distributed under the WU-Minn HCP open access data use term. A multi-shell diffusion scheme was used, and the b-values were 1000, 2000, 3000 s/mm^2^. The number of diffusion sampling directions were 90, 90, and 90, respectively. The in-plane resolution was 1.25 mm. The slice thickness was 1.25 mm. The diffusion data were reconstructed in the MNI space using q-space diffeomorphic reconstruction to obtain the spin distribution function^59^. A diffusion sampling length ratio of 2.5 was used, and the output resolution was 1 mm. The analysis was conducted using the DSI Studio (http://dsi-studio.labsolver.org) and the Connectome Workbench (https://www.humanconnectome.org/software/get-connectome-workbench).

### Diffusion-weighted tractography

Publicly available imaging data from the Human Connectome Project (http://humanconnectome.org) was analyzed with the DSI Studio software (http://dsi-studio.labsolver.org). The HCP 1021 template and ten healthy unrelated control data were analyzed in the present study (Subjects IDs: 102614, 103212, 163129, 304020, 448347, 587664, 990366, 952863, 919966, 996782). Diffusion-weighted tractography was conducted as previously described^6,29^.

The HCP MMP1.0^26^ was originally created in the CIFTI format, which is a surface-based coordinate system (“greyordinates”). Therefore, it is difficult to perform tractography analysis using ROIs created in the CIFTI format^29^. To convert all 180 areas from a surface-based coordinate system to volumetric coordinates, we used the HCP MMP1.0 atlas built in DSI studio software. Segmentation results were visually assessed for accuracy by trained operators. We especially assessed the atlas-based segmentation of DSI studio by confirming the V3A/V3B border is localized at TOS. We also confirmed the fusiform gyrus (VVC and FFC) is segmented between CoS and OTS.

VOF tractography in Fig. 1C was reconstructed as previously described^6^. The fusiform gyrus and the inferior occipital and temporal gyrus were served as the ventral ROI masks. The IPS (intraparietal sulcus) within the occipital lobe and the TOS (transverse occipital sulcus) were served as the dorsal ROI masks. To avoid other fiber contamination, we drew a ROA (region of avoidance) mask on the coronal plane along the splenium of the corpus callosum. For other fasciculi, including AF (arcuate fasciculus) and OR (optic radiation), default parameters in the DSI Studio were used.

To reconstruct fiber tracts of VOF in Figs. 5 and 6, we employed a two-ROI approach. We selected two ROIs as endpoints from three cortical groups consisting of dorsal (e.g., V3A, V3B, V3CD, V6, V6A, V7, IP0), lateral occipital (e.g., LO1, LO2, LO3, V4t, MT, MST), and ventral group (e.g., V4, V8, VMV1, VMV2, VMV3, VVC, FFC, PIT) as previously described^29^. Only superior-inferior projections, which appear as blue projections on RGB map, were selected as candidate fiber tracts of VOF. Then fiber tracts lateral to ILF in the sagittal stratum of Sachs (SSS) were extracted as VOF’s fiber tracts from the candidates.

### Endpoint analysis and Connectogram

Endpoint analysis was performed to quantify the cortical terminations of VOF in tractography using the ‘connectivity matrix’ function in DSI studio, which generates matrices representing the number of fibers terminating within each cortical area. Connectograms that indicate a circular representation of human cortical networks were constructed by on-line CIRCOS software (http://mkweb.bcgsc.ca/tableviewer/)^38,39^. In brief, Circos is a cross-platform Perl application that employs a circular layout to facilitate the display of relationships between pairs of positions by the use of various graphical elements, including ribbons and heat maps. Each cortical area and ribbon were assigned a unique RGB color.

### Laterality index

Lateralization index (LI) was calculated in each case by using the formula: LI = (L−R) / (L + R). L; left, R; right. The LI ranged from −1 (completely right-lateralized) to +1 (completely left-lateralized) as previously reported^60^.

### Statistics

Quantitative data were analyzed by the student *t*-test, with *p* values less than 0.05 considered as statistically significant. Data were expressed as mean ± SEM (standard error of the mean) as indicated.

## Supplementary information

Dataset 1.
