# Supplementary material for: White matter dissection and structural connectivity of the human vertical occipital fasciculus to link vision-associated brain cortex
Source: Sci Rep. 2020 Jan 21;10:820. doi: 10.1038/s41598-020-57837-7 (PMC6972933; doi:10.1038/s41598-020-57837-7)

**White matter dissection and structural connectivity of the human vertical occipital fasciculus to link vision-associated brain cortex**

Tatsuya Jitsuishi**^1^**, Seiichiro Hirono**^2^**, Tatsuya Yamamoto**^1, 3^**, Keiko Kitajo**^1^**, Yasuo Iwadate**^2^**, Atsushi Yamaguchi**^1^** ^*^

Supplementary Information

**
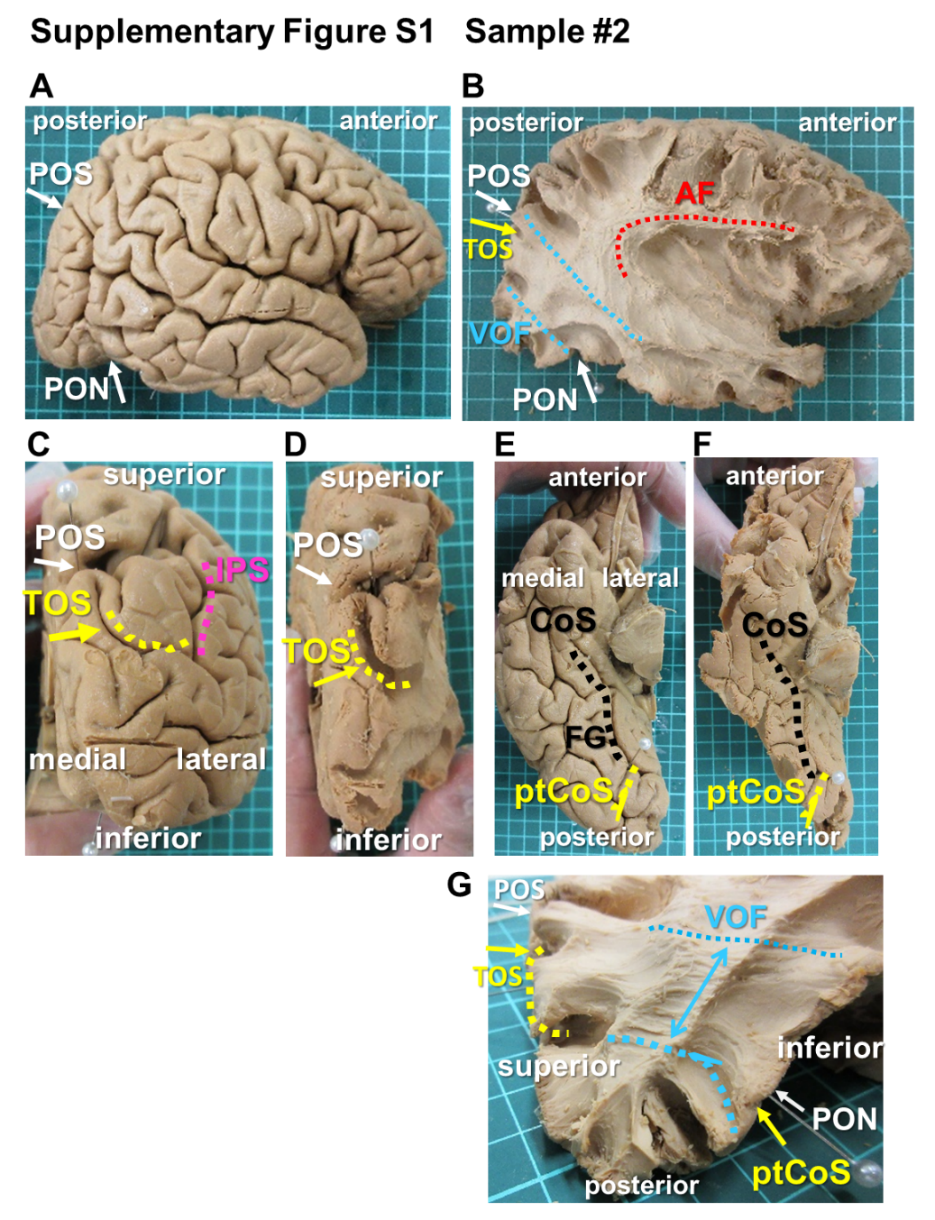
**

**Fig. S1. White matter dissection of the brain (Sample #2)**.

(A) The lateral surface of the hemisphere following the removal of the membranes and vessels.

(B) Removal of frontoparietal and temporal opercula around the insula to expose AF and VOF.

(C)(D) Dissection to expose the dorsal VOF’s cortical projections in TOS.

(E)(F) Further dissection to expose the ventral VOF’s cortical projections in ptCoS.

(G) The magnification of the posterolateral corner of the brain.

AF; arcuate fasciculus, FG; fusiform gyrus, POS; parieto-occipital sulcus, TOS; transverse occipital sulcus, PON; pre-occipital notch, IPS; intraparietal sulcus, CoS; collateral sulcus, ptCoS; posterior transverse CoS.


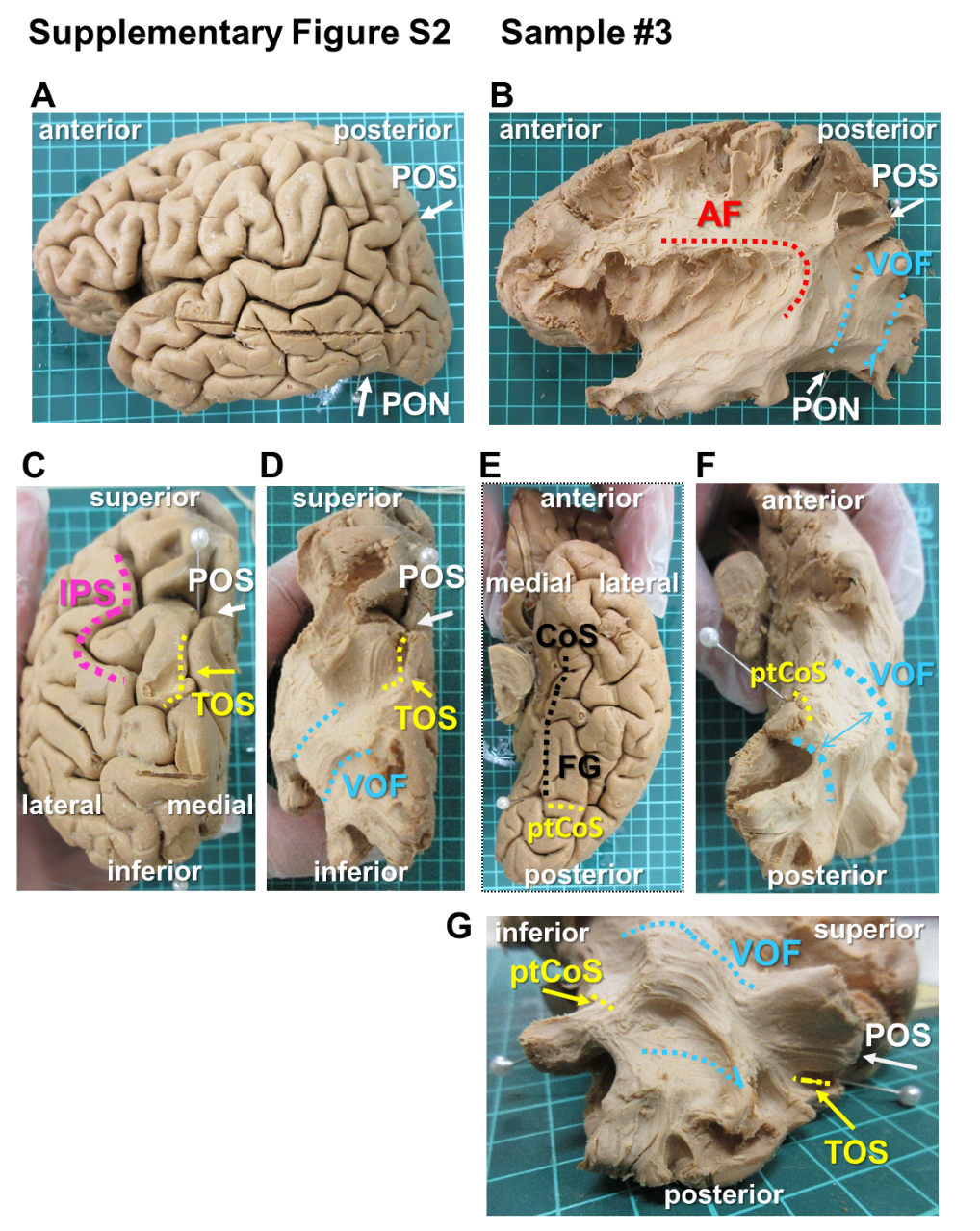


**Fig. S2. White matter dissection of the brain (Sample #3)**.

(A) The lateral surface of the hemisphere following the removal of the membranes and vessels.

(B) Removal of frontoparietal and temporal opercula around the insula to expose AF and VOF.

(C)(D) Dissection to expose the dorsal VOF’s cortical projections into TOS.

(E)(F) Further dissection to expose the ventral VOF’s cortical projections into ptCoS.

(G) The magnification of the posterolateral corner of the brain.

AF; arcuate fasciculus, FG; fusiform gyrus, POS; parieto-occipital sulcus, TOS; transverse occipital sulcus, PON; pre-occipital notch, IPS; intraparietal sulcus, CoS; collateral sulcus, ptCoS; posterior transverse CoS.


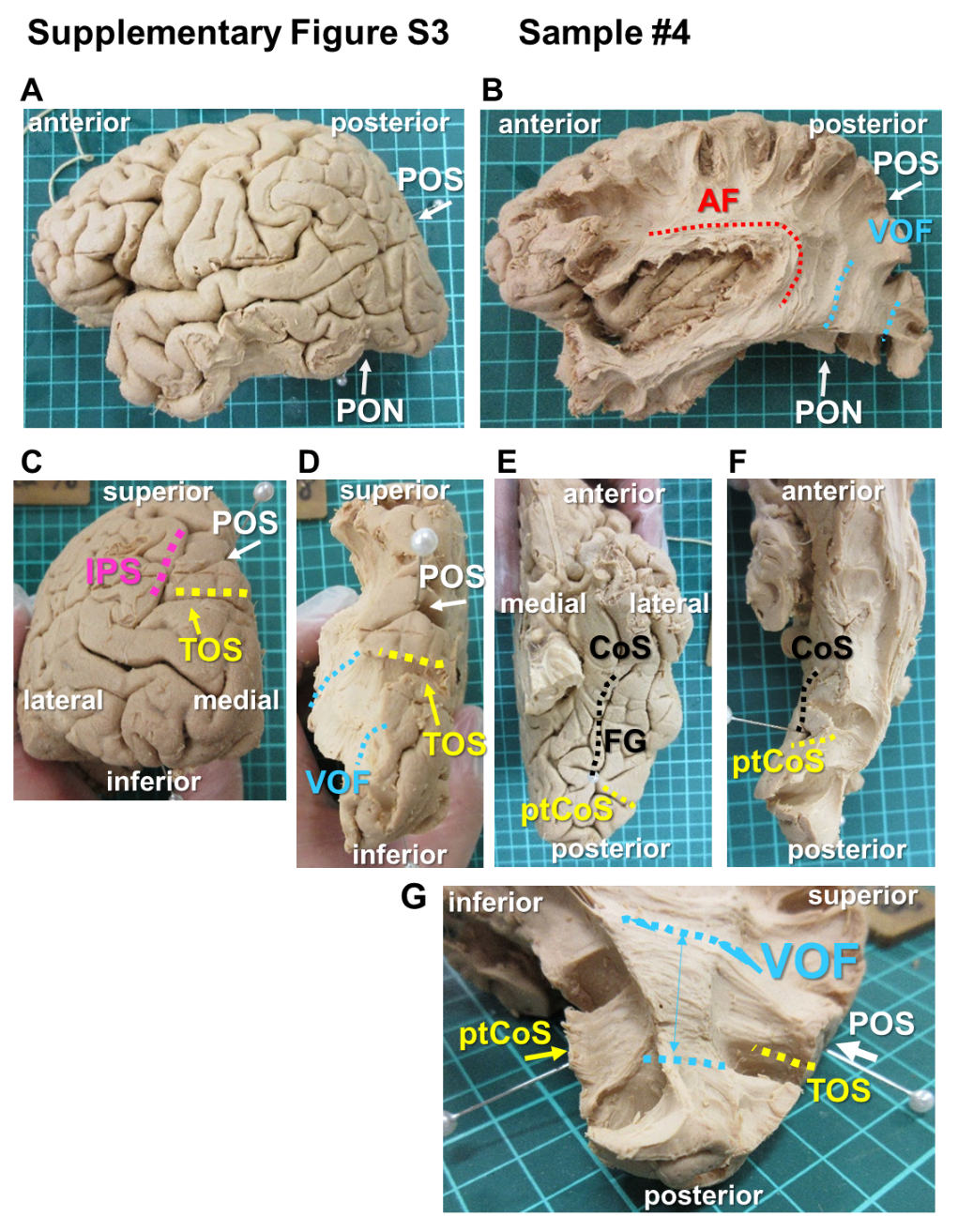


**Fig. S3. White matter dissection of the brain (Sample #4)**.

(A) The lateral surface of the hemisphere following the removal of the membranes and vessels.

(B) Removal of frontoparietal and temporal opercula around the insula to expose AF and VOF.

(C)(D) Dissection to expose the dorsal VOF’s cortical projections into TOS.

(E)(F) Further dissection to expose the ventral VOF’s cortical projections into ptCoS.

(G) The magnification of the posterolateral corner of the brain.

AF; arcuate fasciculus, FG; fusiform gyrus, POS; parieto-occipital sulcus, TOS; transverse occipital sulcus, PON; pre-occipital notch, IPS; intraparietal sulcus, CoS; collateral sulcus, ptCoS; posterior transverse CoS.

**Supplementary Figure S4**


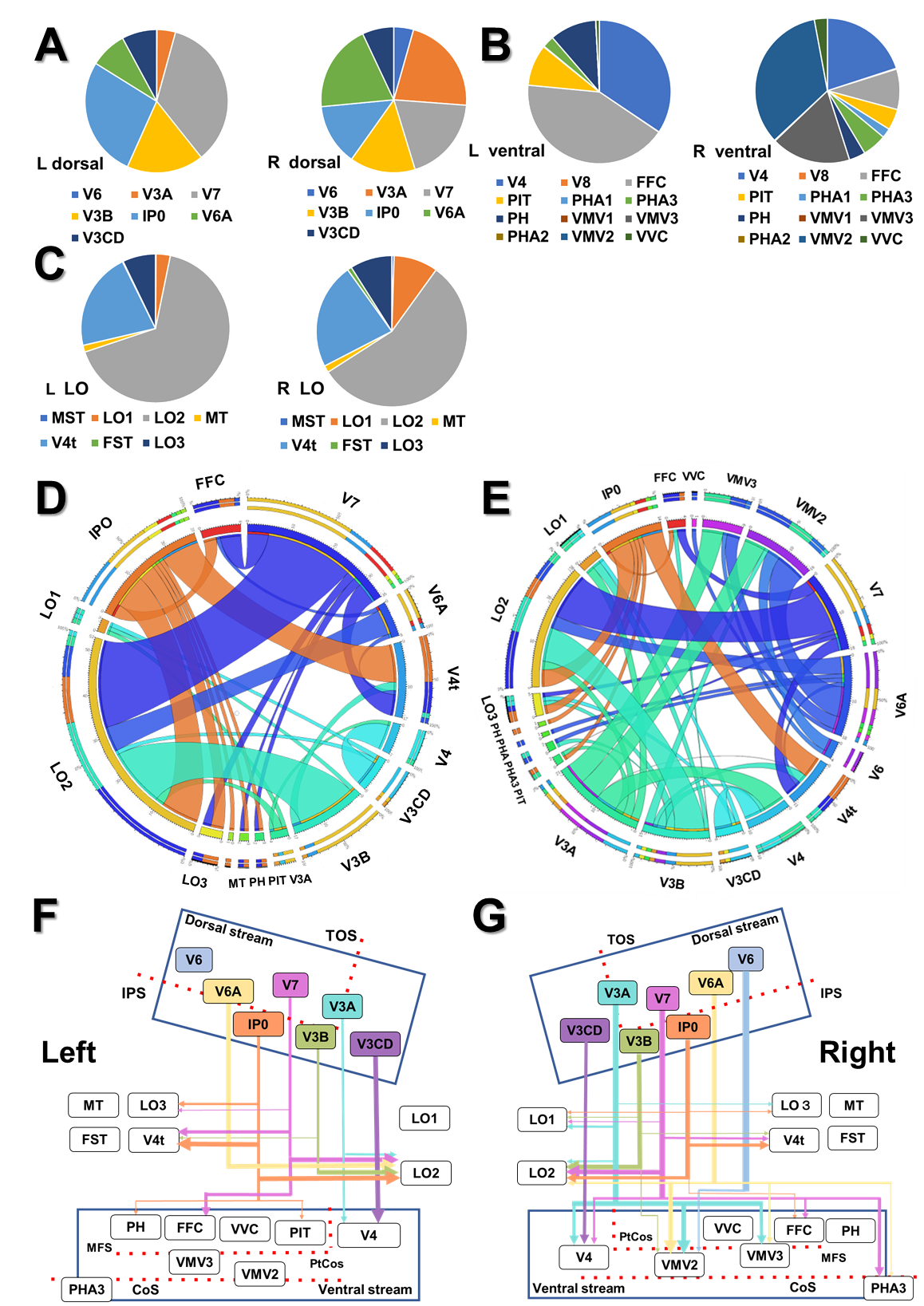


**Figure S4. Endpoint analysis, Connectogram, and Schematic diagram for the connectivity patterns of VOF in the HCP-1021 template.**

(A)(B)(C) The ratio (%) of cortical areas in which VOF’s cortical endpoints fall on dorsal (A), ventral (B), and lateral occipital (C) visual cortex in the left and the right hemisphere, respectively. Dorsal, dorsal visual cortex; Ventral, ventral visual cortex; LO, lateral occipital visual cortex; L, left; R, right.

(D)(E) Connectograms indicating the connectivity patterns of VOF in the left (D) and the right (E) hemisphere, respectively. Circular color maps detail the scale for each connection. The outermost two rings reflect the various cortical areas of the HCP MMP 1.0 atlas. The length of arc reflects the count of tracts terminating within each cortical area. The ribbon size represents the computed degrees of connectivity (i.e., the number of tracts) between segmented brain regions. MT, middle temporal; PHA, parahippocampal area; PIT, posterior inferior temporal; LO, lateral occipital; IP0, intraparietal 0; FFC, fusiform face complex; VVC, ventral visual complex; VMV, ventromedial visual area. The original versions of these connectograms are shown in the Supplementary Fig. S5 and S6.

(F)(G) Schematic diagrams of the structural connectivity for VOF’s fiber tracts in the left (F) and the right (G) hemisphere. The arrow size reflects the computed degrees of connectivity (i.e., the number of tracts) between segmented brain regions. 　TOS, transverse occipital sulcus; IPS, intraparietal sulcus; CoS, collateral sulcus; ptCoS, posterior transverse CoS; MFS, middle fusiform sulcus.

**Supplementary Figure S5**


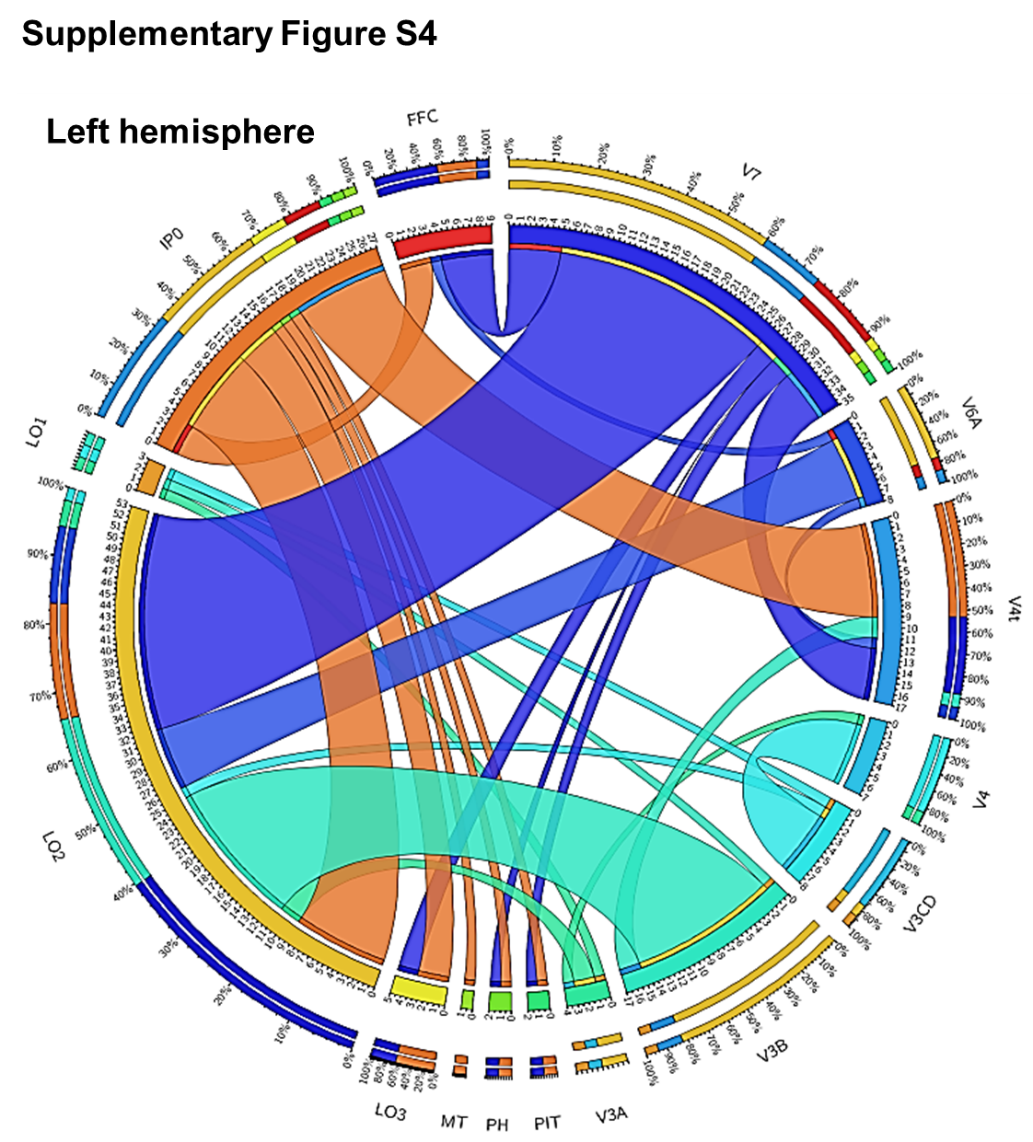


**Fig. S5. Connectogram for the connectivity patterns of the VOF in the left hemisphere of HCP1021 template.**

A circular color map details the scale for each connection. The outermost two rings show the various cortical areas of the HCP MMP 1.0 atlas. The length of arc for each cortical area (noted as 0-100 %) reflects the count of fiber tracts terminating within each cortical area. The ribbon size represents the computed degrees of connectivity (i.e., the number of tracts) between segmented brain regions. The number on the most inward ring reflects the computed total count of each fiber tracts. MT, middle temporal; PHA, parahippocampal area; PIT, posterior inferior temporal; LO, lateral occipital ;IP0, intraparietal 0 ; FFC, fusiform face complex; VVC, ventral visual complex; VMV, ventromedial visual area.

**Supplementary Figure S6**


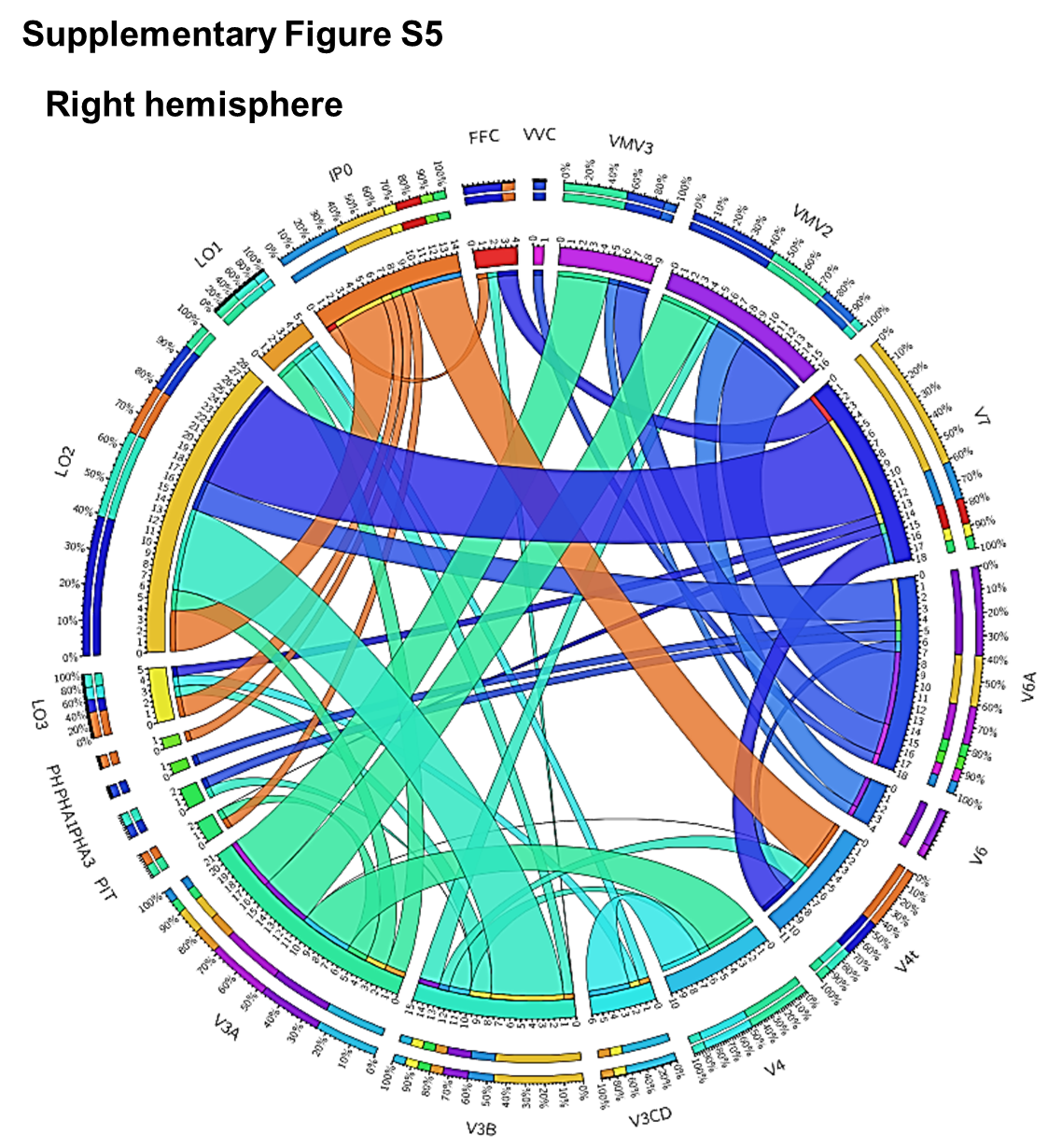


**Fig. S6. Connectogram for the connectivity patterns of the VOF in the right hemisphere of HCP1021 template.**

A circular color map details the scale for each connection. The outermost two rings show the various cortical areas of the HCP MMP 1.0 atlas. The size of arc for each cortical area (noted as 0-100 %) reflects the count of fiber tracts terminating within each cortical area. The ribbon size represents the computed degrees of connectivity (i.e., the number of tracts) between segmented brain regions. The number on the most inward ring reflects the computed total count of each fiber tracts. MT, middle temporal; PHA, parahippocampal area; PIT, posterior inferior temporal; LO, lateral occipital ;IP0, intraparietal 0 ; FFC, fusiform face complex; VVC, ventral visual complex; VMV, ventromedial visual area.

**Supplementary Figure S7**


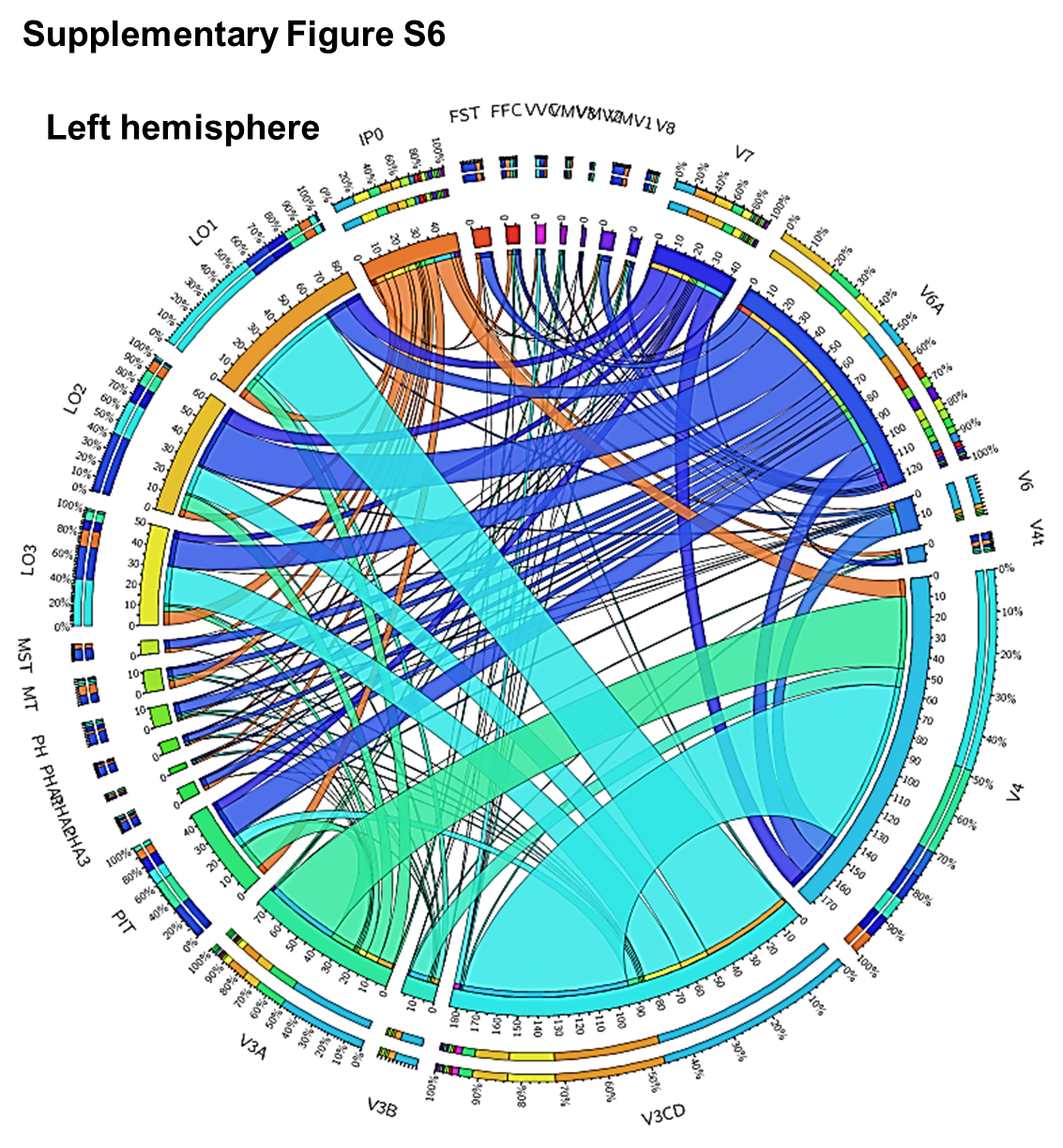


**Fig. S7. Connectogram for the connectivity patterns of the VOF in the left hemisphere of individual brains.**

A circular color map details the scale for each connection. The outermost two rings show the various cortical areas of the HCP MMP 1.0 atlas. The size of arc for each cortical area (noted as 0-100 %) reflects the count of fiber tracts terminating within each cortical area. The ribbon size represents the computed degrees of connectivity (i.e., the number of tracts) between segmented brain regions. The number on the most inward ring reflects the computed total count of each fiber tracts. MT, middle temporal; PHA, parahippocampal area; PIT, posterior inferior temporal; LO, lateral occipital ;IP0, intraparietal 0 ; FFC, fusiform face complex; VVC, ventral visual complex; VMV, ventromedial visual area.

**Supplementary Figure S8**


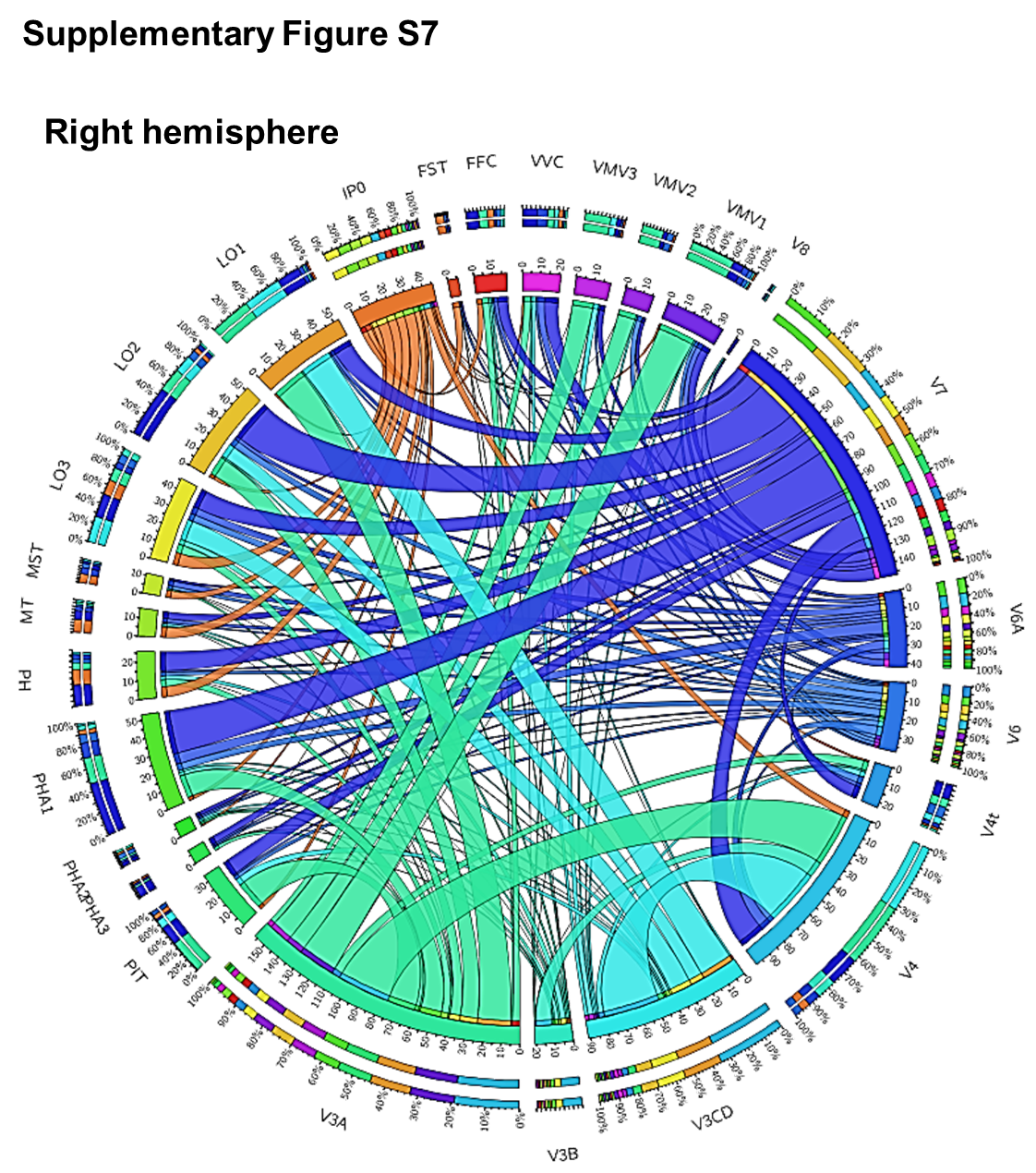


**Fig. S8. Connectogram for the connectivity patterns of the VOF in the left hemisphere of individual brains.**

A circular color map details the scale for each connection. The outermost two rings show the various cortical areas of the HCP MMP 1.0 atlas. The size of arc for each cortical area (noted as 0-100 %) reflects the count of fiber tracts terminating within each cortical area. The ribbon size represents the computed degrees of connectivity (i.e., the number of tracts) between segmented brain regions. The number on the most inward ring reflects the computed total count of each fiber tracts. MT, middle temporal; PHA, parahippocampal area; PIT, posterior inferior temporal; LO, lateral occipital ;IP0, intraparietal 0 ; FFC, fusiform face complex; VVC, ventral visual complex; VMV, ventromedial visual area.

**Supplementary Table 1**


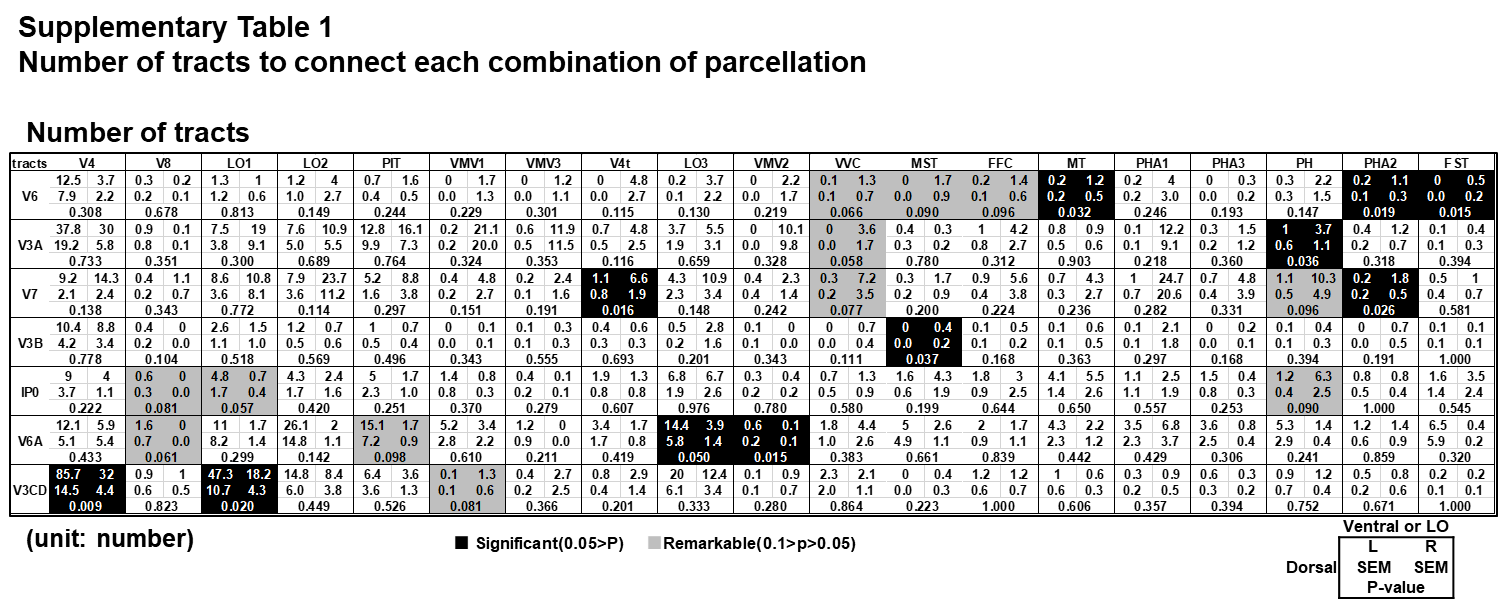
Number of tracts to connect each combination of cortical area

**Supplementary Table 2**

Volume of tracts to connect each combination of cortical area


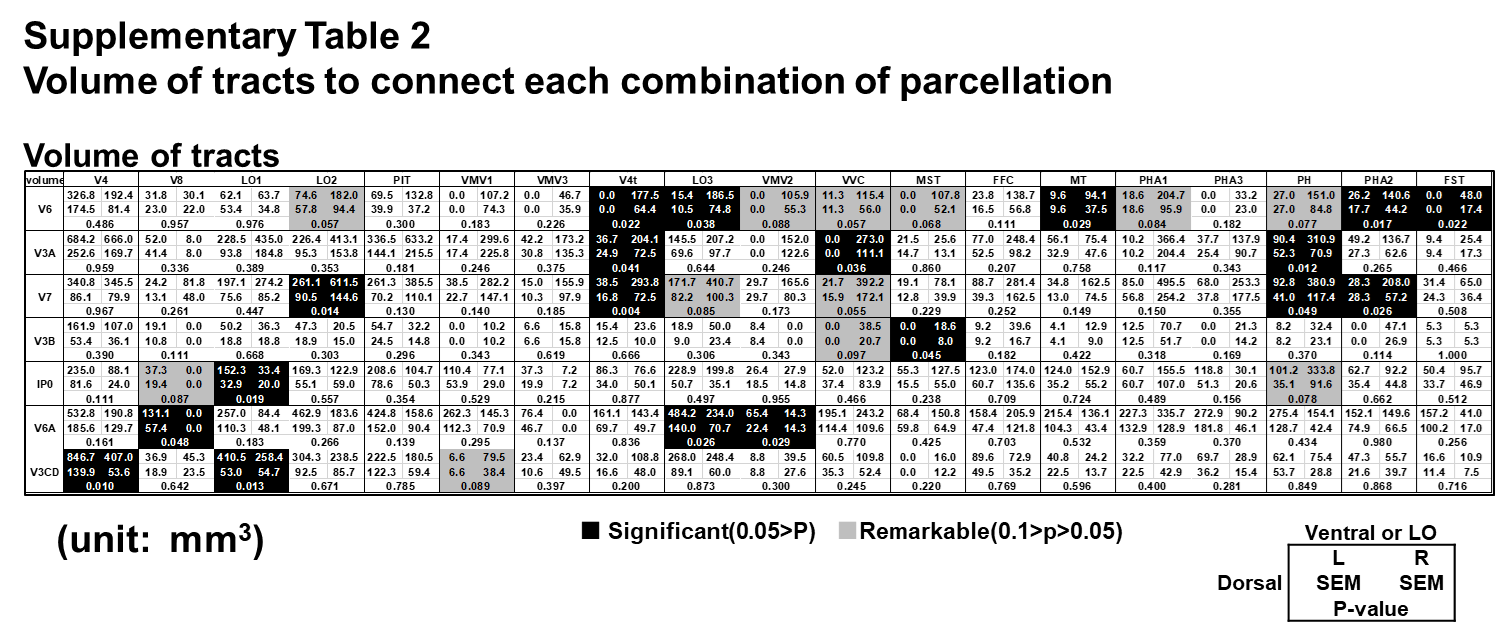


**Supplementary Table 3**

Fractional anisotropy (FA) of tract to connect each combination of cortical area


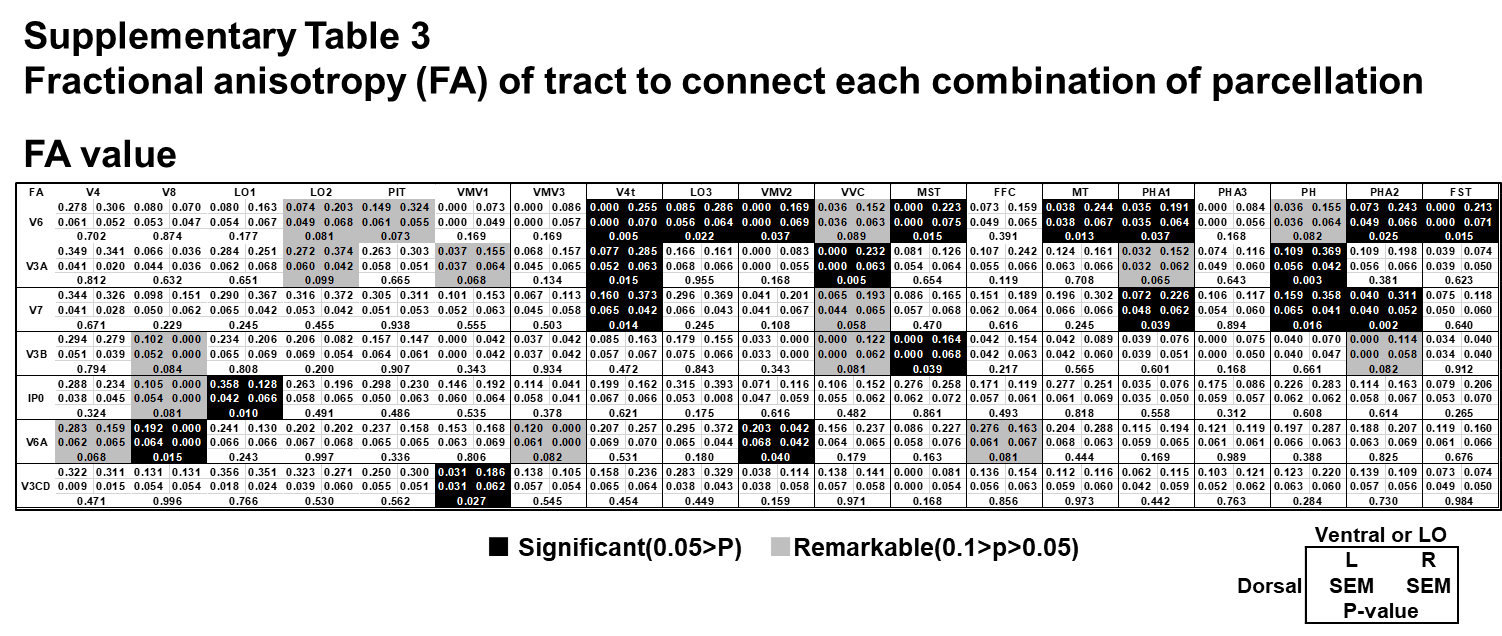


**Supplementary Table 4**

Laterality of the tract to link each combination of cortical area


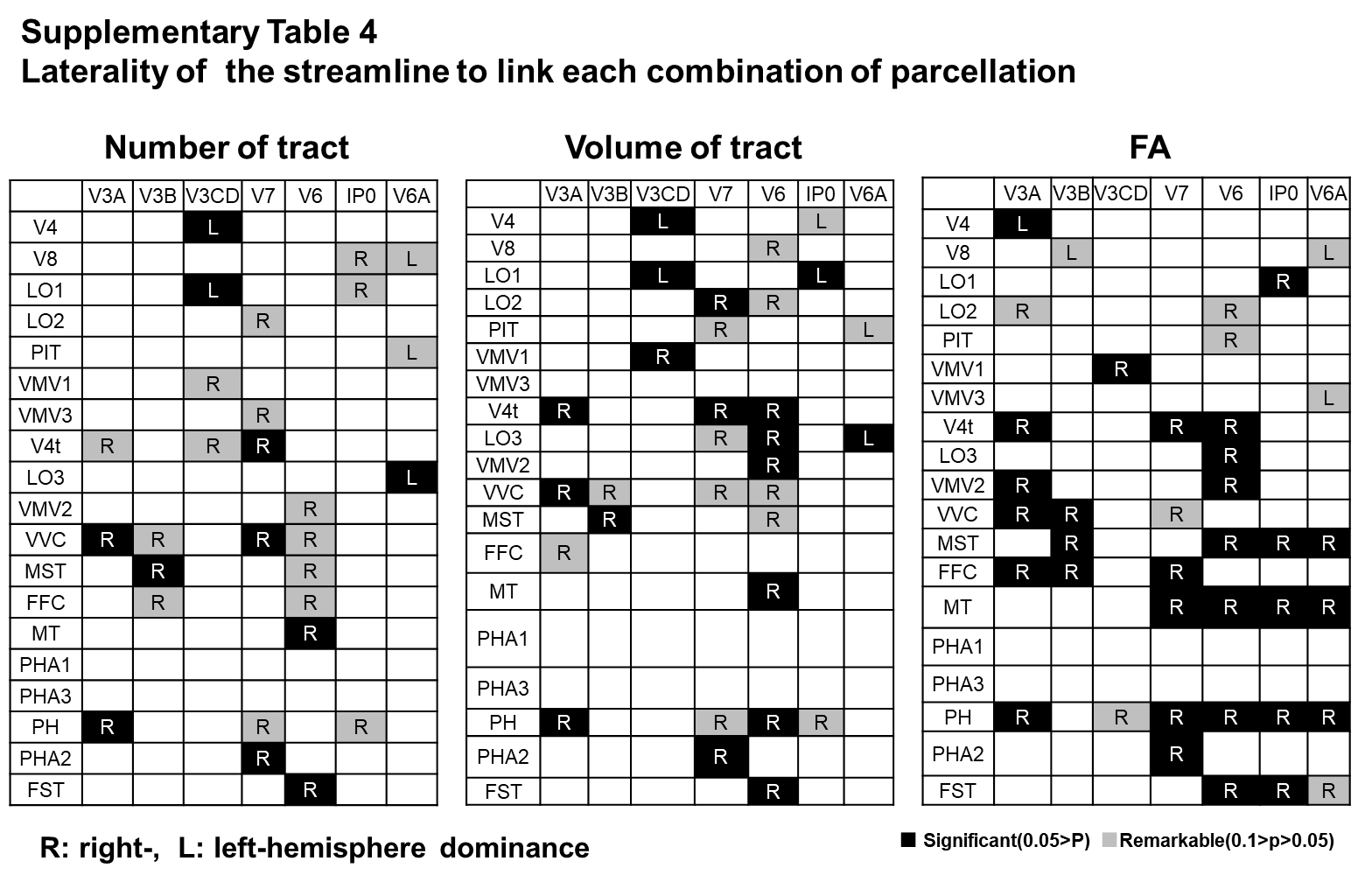

Supplement: Supplementary file 1 — Dataset 1. [file 41598_2020_57837_MOESM1_ESM.docx]
